# Aberrant miR-874-3p/leptin/EGFR/c-Myc signaling contributes to nasopharyngeal carcinoma pathogenesis

**DOI:** 10.1186/s13046-022-02415-0

**Published:** 2022-07-01

**Authors:** Sheng-Dean Luo, Hsin-Ting Tsai, Chung-Feng Hwang, Tai‐Jan Chiu, Shau‐Hsuan Li, Ya-Ling Hsu, Chang-Chun Hsiao, Chang-Han Chen

**Affiliations:** 1 Department of Otolaryngology, Kaohsiung Chang Gung Memorial Hospital, Chang Gung University College of Medicine, Kaohsiung, 833 Taiwan; 2 Graduate Institute of Clinical Medical Sciences, College of Medicine, Chang Gung University, Taoyüan, 33302 Taiwan; 3 Department of Medical Research, Chung Shan Medical University Hospital, Taichung City, 40201 Taiwan; 4 Department of Hematology-Oncology, Kaohsiung Chang Gung Memorial Hospital and Chang Gung University College of Medicine, Kaohsiung, 833 Taiwan; 5 Division of Pulmonary and Critical Care Medicine, Kaohsiung Chang Gung Memorial Hospital and Chang Gung University College of Medicine, Kaohsiung, 83301 Taiwan; 6 Institute of Medicine, Chung Shan Medical University, No.110, Sec.1, Jianguo N. Rd, Taichung City, 40201 Taiwan

**Keywords:** Leptin, NPC, EGFR

## Abstract

**Background:**

Leptin is important in physiological and pathological functions in various cancers, however, the significance and mechanisms of leptin in nasopharyngeal carcinoma remain ambiguous.

**Methods:**

Leptin expression was analyzed by QPCR, immunohistochemistry, Western blotting, and TCGA database. The impact of gain- or loss-of-function of leptin were determined by MTT, colony formation, wound healing, and Transwell assays in NPC cells, and by a xenograft tumor model. Leptin-modulated glucose consumption and lactate production were assessed by ELISA. Furthermore, leptin-regulated signaling pathways were examined by QPCR and Western blotting assays. The immunoprecipitation assay was conducted to determine interaction between leptin and EGFR. In addition, miR-874-3p-regulated leptin expression was evaluated using bioinformatics, QPCR, luciferase assay, AGO2-RIP assay, and Western blotting.

**Results:**

In this study, we found that leptin was highly expressed in the sera and tumor tissues of patients with NPC, and elevated leptin expression was associated with advanced clinical features and poor prognosis. Functional assays demonstrated that leptin remarkably promoted NPC cell growth, motility, and glycolysis in vitro and in vivo. Mechanistically, leptin associated with EGFR, resulting in enhanced cell growth through the regulation of cell-cycle related markers, glycolysis-related genes, and EGFR/AKT/c-Myc signaling. Moreover, leptin potentiated the invasive capacity of NPC cells by promoting EMT. We further explored that miR-874-3p influenced leptin-mediated NPC progression. Overexpression of miR-874-3p prevented cell growth, motility, glucose consumption, and lactate production in NPC cells, whereas miR-874-3p inhibition had the opposite effects. AGO-RIP assays confirmed that Argonaute 2 (AGO2), a protein associated with miR-874-3p, regulated leptin expression in NPC cells. The rescue assays indicated that inhibition of leptin suppressed the effects of miR-874-3p inhibitor. In clinical specimens, miR-874-3p was negatively correlated with leptin.

**Conclusions:**

Leptin may serve as a novel prognostic factor and potential therapeutic target for patients with NPC. In addition, a newly discovered regulatory axis of leptin/EGFR/AKT/c-Myc can provide a novel therapeutic strategy for NPC.

**Supplementary Information:**

The online version contains supplementary material available at 10.1186/s13046-022-02415-0.

## Background

Nasopharyngeal carcinoma (NPC) is a malignant tumor involving the head and neck areas. Unlike other head and neck cancers, NPC is characterized by increased invasiveness and metastasis [1]. Survival outcomes are significantly better in patients with early-stage NPC than in those with late-stage NPC [1, 2]. Although NPC is rare in most parts of the world, it has a high regional incidence in Asia, with that in Southern China and Southeast Asia ranking among the highest in the world [2]. Generally, there are three main types of NPC according to the World Health Organization (WHO), which include keratinizing squamous cell carcinoma, non-keratinizing carcinoma and undifferentiated carcinoma [3]. Epstein-Barr virus (EBV) has been shown to be a risk factor for NPC, especially for non-keratinizing and undifferentiated carcinoma [3–5]. The earliest evidence of NPC associated with EBV was identified in 1973 [6]*.* Although EBV is detected in most patients with NPC, cases of EBV-negative NPC have also been reported [4, 7]. Other lifestyle-associated risk factors and/or host genetic variants may interact with EBV to play roles in the carcinogenesis of NPC [5, 8, 9]. Obesity can occur due to unhealthy lifestyle habits and has been associated with an increased risk for various cancers, including breast, esophageal, pancreatic, and colorectal cancers [10–13]. Interestingly, several epidemiological studies have shown that patients with NPC have higher BMI, implying that overweight or obese people are tended to have a greater risk of NPC [14–16]. However, it remains unclear whether elevated leptin levels*,* which are commonly found in the obese population [17], play a role in the carcinogenesis of NPC.

Leptin is a hormone secreted by adipocytes [17]. It acts in an autocrine and/or paracrine manner to control and coordinate several biological and pathological activities throughout the body by binding to the leptin receptor [18, 19]. Accumulating evidence suggests that leptin contributes to various aspects of tumor progression and metastasis in multiple cancers such as breast, oral, and pancreatic cancers [20, 21]. In addition, leptin attenuates the outcome of cancer therapies and thus promotes tumor progression by inducing expression of factors such as ACTC1 and Notch [21]. The results of these studies underline the importance of understanding the mechanisms of leptin in tumor progression to improve the treatment of patients with cancer. With regards to NPC, only a limited number of studies were conducted to determine if leptin directly plays a role in modulating tumor progression in NPC. A recent genome-wide association study demonstrated that leptin is potentially responsible for regulating cell growth leading to human NPC development [22], suggesting that leptin may represent a key to understand the etiology and progression of NPC. Thus, to understand if leptin is a key player in the tumor progression of NPC, we conducted a clinical study and biochemical approaches to determine if leptin levels have a causal link to NPC.

MicroRNAs (miRNAs or miRs), well-known regulators in tumor progression and metastasis of various cancers [23–25], are attractive options for NPC therapy. These non-coding single-stranded RNAs regulate the expression of their targeted genes post-transcriptionally by typically binding to untranslated region (3′UTR) regions in mRNAs [25]. In NPC, miR-138 and miR-184, suppress tumor progression by targeting CCDN1 and Notch2, respectively [26, 27]. In contrast, miR-214 and *miR*-141-3p, which are known as oncogenic miRNAs, have been shown to promote cell proliferation and facilitate tumor progression of NPC [25, 28]. Recent studies have demonstrated that miR-874-3p is an important negative regulator of cancers such as epithelial ovarian, esophageal squamous cell carcinoma and hepatocellular carcinoma [29–31], indicating that miR-874-3p plays a significant role in modulating tumor progression. However, neither the role of miR-874-3p in NPC nor its regulatory function in leptin gene expression has been studied.

In this study, we found that patients with NPC had higher levels of leptin in serum compared to that of healthy groups and had elevated leptin in the tumor compared to adjacent non-tumor tissues. Most importantly, we found that increased leptin expression in NPC was associated with advanced cancer stages and poor patient survival. Functional studies indicated that leptin promoted proliferation, invasion, as well as glycolysis for NPC progression both in vitro and in vivo. Additionally, miR-874-3p inhibited the expression of *leptin* mRNA and impaired the leptin-elicited aggressive phenotypes of NPC. Our findings provide insights into the mechanisms of leptin-driven carcinogenesis in NPC and the potential of leptin as a therapeutic target for NPC.

## Methods

### Clinical samples

A total of 50 paired specimens of NPC from the Tissue Bank of Chang Gung Memorial Hospital obtained between June 2017 and September 2021 were included in this study. Fresh blood samples of 20 patients with NPC and 17 health volunteers were collected. This study was approved by Chang Gung Memorial Hospital, and informed consent was obtained from all participants. The pathological stage and nodal status were obtained from the primary pathology reports. All clinicopathological features were defined according to the classification guideline of the American Joint Committee on Cancer (AJCC).

### Cell culture

NPC cell lines, NPC-TW06 and NPC-TW02 (provided by Dr. Chin-Tarng Lin, National Taiwan University, Taiwan), were grown in DMEM (Gibco, USA) containing 2 mM L-glutamine, 3.7 g/L sodium bicarbonate, MEM non-essential amino acids, 10% FBS, and 100 U/ml penicillin and streptomycin (Gibco, USA). All the cells were incubated in a humidified atmosphere of 5% CO_2_ at 37° C.

### RNA extraction and Real-time quantitative RT-PCR

Total RNA was extracted using TRIzol following the manufacturer's instructions. Isolated RNA was reverse transcribed into cDNA using the PrimeScript RT Master Mix Kit (TAKARA). RT-PCR was performed using SYBR Green Master Mix on the Roche Real-time PCR system. GAPDH was used as an internal control. The relative gene expression levels were normalized to human GAPDH levels and calculated using the comparative Ct (2-^ΔΔCT^) method. The primers of target mRNA/miRNA and internal control for QPCR were described as following: leptin forward, 5’-GAAGACCACATCCACACACG-3’; leptin reverse, 5’-AGCTCAGCCAGACCCATCTA-3’; EGFR forward, 5ʹ- ACATTAAGGAGGCCTGTCT-3’; EGFR reverse, 5ʹ- AGCAAACTTGTACCAGCTT-3’; TPI1 forward, 5’-CCCAGGAAGTACACGAGAAG-3’; TPI1 reverse, 5’-CAGTCACAGAGCCTCCATAAA-3’; ALDOA forward, 5’-GCAACTTTCCTCTGCCTAGC-3’; ALDOA reverse, 5’-AAGCAGAGACAGTTGAGGCT-3’; ENO1 forward, 5’-GGGAATCCCACTGTTGAGGT-3’; ENO1 reverse, 5’-CGGAGCTCTAGGGCCTCATA-3’; HK1 forward, 5’-GCTCTCCGATGAAACTCTCATAG-3’; HK1 reverse, 5’- GGACCTTACGAATGTTGGCAA-3’; PFKL forward, 5’-CTACGAGGGCTATGAGGGC-3’; PFKL reverse, 5’- GATGACGCACAGGTTGGTGA-3’; GAPDH forward, 5’-GCACCGTCAAGGCTGAGAAC-3’; GAPDH reverse, 5’- TGGTGAAGACGCCAGTGGA-3’; hsa-miR-874-3p forward, 5’- GAACTCCACTGTAGCAGAGATGGT-3’; and hsa-miR-874-3p reverse, 5’-CATTTTTTCCACTCCTCTTCTCTC-3’ [30].

### Plasmid transfection, siRNA knockdown, and miRNA mimic/inhibitor experiments

The expression plasmids encoding leptin (pCMV6-Flag-Lep) or a control empty vector (pCMV6-Flag) with a C-terminal Myc-DDK tag were purchased from OriGene Technologies. Plasmids were transiently transfected into TW06 and TW02 cells using Lipofectamine 3000 (Invitrogen, USA) according to the manufacturer’s instructions. Two different synthetic interfering RNAs (siRNAs) that target leptin (leptin siRNA, Sigma-Aldrich Co., NM_000230) and has-miR-874-3p mimic / inhibitor (Sigma-Aldrich Co., HMI0926 / HSTUD0926) were transiently transfected into TW06 and TW02 cells using Lipofectamine RNAi MAX (Invitrogen, USA) according to the manufacturer’s instructions. The siRNA sequences of leptin were 5’- GGAACUCUGGCUUCCAGGU-3’ and 5’-CUGACUCCUCUAAGCCACU-3’. The sequences of miR-874-3p mimic and miR-874-3p inhibitor were 5’- CUGCCCUGGCCCGAGGGACCGA-3’ and 5’- CUGCCCUGGCCCGAGGGACCGA-3’.

### Immunoblot analysis

Cell samples were homogenized in a RIPA buffer (Thermo Fisher Scientific, USA) containing protease and phosphatase inhibitor (Merck Millipore). The protein concentration in each sample were estimated using a Bio-Rad protein assay (Bio-Rad Laboratories, USA). Immunoblotting was performed with an antibody against DDK (FLAG tag) (1:1000, TA50011) purchased from OriGene Technologies. Antibody against β-actin (1:20,000, A5541) was purchased from Sigma-Aldrich. Antibodies against leptin (1:1000, ab3583), vimentin (1:4000, ab92547), and c-MYC (1:1000, ab32072) were purchased from Abcam. Antibodies against p-AKT (1:2000, #9271), AKT (1:1000, #9272), p-mTOR (1:1000, #2971), mTOR (1:2000, #2972), p-ERK (1:3000, #9101), ERK (1:6000, #4695), p-EGFR (1:1000, #3777), and EGFR (1:2000, #4267) were purchased from Cell Signaling Technology Inc. Antibodies against N-cadherin (1:500, sc-7939), cyclin D1 (1:500, sc-717), cyclin E (1:500, sc-247), CDK4 (1:500, sc-23896), and p27 (1:250, sc-528) were purchased from Santa Cruz Biotechnology Inc. Antibodies against ZO-1 (1:1000, A0659), ZEB1 (1:1000, A5600), Claudin-1 (1:1000, A2196) were purchased from ABclonal Inc. Antibodies against E-cadherin (1:4000, GTX124178), and p21 (1:1000, GTX62525) were purchased from GeneTex Inc. Horseradish peroxidase-conjugated goat anti-rabbit and goat anti-mouse antibodies (PerkinElmer Inc.) were used. Signals were revealed using Immobilon Western Chemiluminescent HRP Substrate (Merck Millipore).

### Immunohistochemical staining

Sections from human NPC tissues and the mice tumors were treated with 3% H_2_O_2_ for 30 min for antigen retrieval. Sections were incubated with a primary antibody specifically against leptin (1:500, GeneTex Inc109204). Antibody against c-MYC (1:100, ab32072) was purchased from Abcam Inc. Antibodies against p-ERK (1:500, #9101), ERK (1:500, #4695), p-EGFR (1:400, #3777), EGFR (1:500, #4267), and ki67 (1:100, #9449) were purchased from Cell Signaling Technology Inc. Sections were incubated overnight at 4 °C. Sections were then washed in phosphate buffered saline with 0.5% Tween-20 (PBST), and incubated with a peroxidase-labeled polymer conjugated to goat anti-mouse or anti-rabbit IgG as secondary antibody for 30 min (Agilent Technologies, Inc., DaKo Real EnVision Detection SystemsPeroxidase/DAB, Rabbit/Mouse). The staining was visualized with 3, 3'-diaminobenzidine (DAB) as chromogen and slides were counterstained with hematoxylin (Sigma-Aldrich Co.), dehydrated, and mounted.

### Cell proliferation assay

Cell viability was determined by an MTS (Promega Co.) assay. After transfection, cells were implanted at a density of 2 × 10^3^ cells per well in 96-well plates (Corning Inc.) for 24, 48 and 72 h. MTS was added to each well and incubated for 2 h. The optical density (OD) was determined with a microplate reader (Molecular Devices) at 490 nm. Each experiment was performed in triplicate.

### Colony formation assay

On the first day after transfection, cells (500 cells/well) were plated in 6-well plates, and incubated for 14 days. Cell colonies were stained with crystal violet (Sigma-Aldrich Co.) for 10 min at room temperature. The colony number was counted using ImageJ software.

### Wound healing migration assay

The cell migration assay was performed using a wound healing assay. After transfection, cells were seeded in ibidi Culture-Inserts (ibidi, USA) and cultured overnight. Transfected confluent cells were changed into medium containing 1% FBS at the start of the assay. The cell migration status toward the gap area was photographed (× 100) (Olympus America Inc.).

### Transwell invasion assay

The invasion chamber with 8 µm pore-size PC membranes (Thermo Scientific™ Nunc™) were covered with 75 µL of 0.3 mg/mL matrigel (Corning Inc.) and then incubated at 37 °C overnight. After transfection, cells were seeded in the upper chambers with medium containing 1% FBS and 0.6 mL medium supplemented with 20% FBS was added to the lower chambers. The invasive ability of cells was determined at 24 h. Cells were fixed using methanol for 10 min and stained with crystal violet (Sigma-Aldrich Co.) for 10 min at room temperature. The non-migrating cells were scraped with cotton swabs. Images were taken using an inverted microscope (× 200) (Olympus America Inc.).

### Glucose consumption, and lactate production

The concentrations of glucose and lactate in the culture media were measuredd by glucose colorimetric assay and lactate colorimetric assay kits (BioVision Research Products) respectively according to the manufacturer’s instructions. Briefly, cells were seeded in 6-well plates and cultured in phenol-red free DMEM for 48 h. The cultured medium was mixed with the reaction solution. Glucose and lactate levels were measured at 450 nm using a microplate reader.

### ELISA

The protein levels of leptin in sera of patients with NPC and healthy groups were assessed using human leptin ELISA KIT (Biovision). The procedures were performed in accordance with the manufacturer’s recommendations. A generated standard curve was generated to allow for the assessment of leptin concentration in the analyzed samples.

### Dual-luciferase reporter assay

The 3’UTR of wild type or mutant leptin, which is predicted to harbor the miR-874-3p binding site, was constructed into the pmirGLO luciferase vector (Promega, Madison, WI). Cells were co-transfected with miR-874-3p mimic (or negative control) and the wild-type or mutated leptin reporter by Lipofectamine 2000. After transfection 48 h, the luciferase activities were determined by using a dual-luciferase reporter system (Promega) according to the manufacturer’s instruction. All reporter gene assays were performed in triplicate and repeated at least 3 times.

### RNA immunoprecipitation assay

The Magna RIP kit (Millipore, USA) was used to conduct RIP experiments according to the manufacturer’s instructions. Briefly, cells were collected and lysed in RIP buffer. Then, antibodies against AGO2 or IgG were added to the extracts, and incubated with magnetic bead-antibody complexes for 4 h at 4 °C. Thereafter, the RNA was purified, and qRT-PCR analysis was used for determining the relative expression of miRNA-874-3p and leptin.

### Co-immunoprecipitation

Cells were lysed at 4 °C in an immunoprecipitation lysis buffer containing protease inhibitor cocktail (Roach, USA). The proteins were incubated with antibody for 16 h at 4 °C followed by a 2 h incubation with Protein A/G (Pierce, MA) at 4 °C. After three washes with IP lysis buffer, protein samples were collected by boiling in 1 × SDS loading buffer and subjected to standard SDS-PAGE and western blotting.

### Xenograft studies

After generating stably transfected shNC and shLeptin cell lines, the cells (1 × 10^7^ shNC or shLeptin cells) were subcutaneously injected into the dorsal flanking sites of male NOD SCID mice (*N* = 4 in shNC group, *N* = 5 in shLeptin group, 4 weeks). The mice were sacrificed using 5% isoflurane (Piramal Critical Care Inc.), and the tumors were weighed and excised. The tumor size and body weight were measured twice per week. Tumor volumes were determined by external measurements and calculated according to the equation, V = (W^2^ × L)/2 (V = volume, L = length and W = width). The mice were sacrificed after 50 days and tumor weights were measured. All procedures were approved by the guidelines of the Institutional Animal Care and Use Committee at Kaohsiung Chang Gung Memorial Hospital.

### Statistical analysis and software

Statistical analysis was performed using GraphPad Prism 8 (GraphPad Software) on n ≥ 3 biological replicates, and presented as mean ± SD or mean ± SEM. Comparisons of the groups were analyzed by Student's t-test or one-way ANOVA. *P*-values less than 0.05 were considered significant. *Denotes a *p*-value less than 0.05; **denotes a *p*-value less than 0.01; and ***denotes a *p*-value less than 0.001.

## Results

### Leptin is up-regulated in human NPC tissues and correlates with poor prognosis

**Fig. 1:**
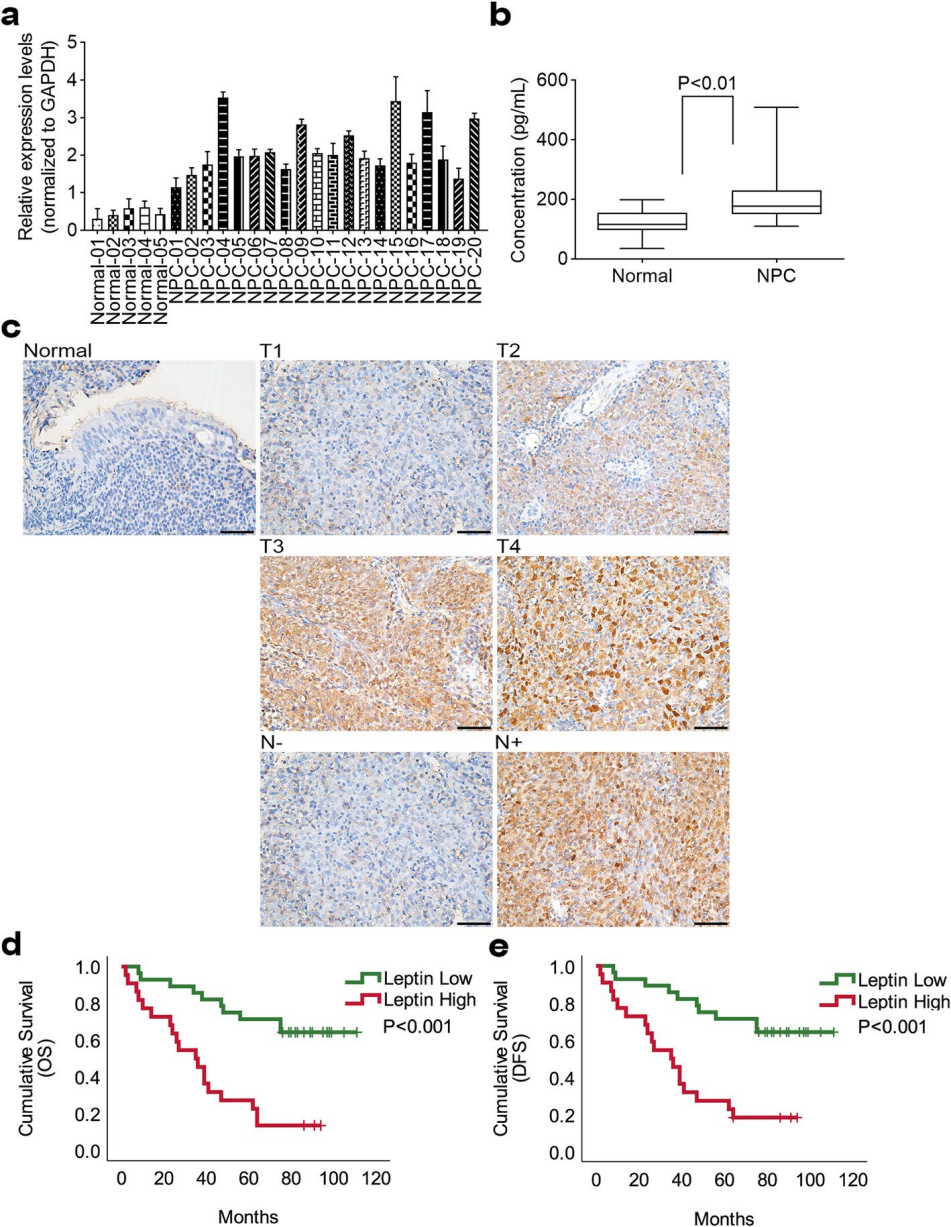
Leptin is overexpression in nasopharyngeal carcinoma. **a** The mRNA expression level of leptin was determined in 5 benign nasopharyngeal inflammatory tissues and 20 NPC tissues. **b** Serum level of leptin in NPC patients and healthy controls were detected by ELISA. **c** Leptin expression was determined by immunohistochemistry in 50-paired paraffin-embedding NPC tissues and adjacent non-tumor tissues, representative images of pathological tumor stage (T) and lymph node stage (N). **d** and **e** Kaplan–Meier survival analysis and log-rank tests indicate that high expression level of leptin was associated with poor OS and DFS in NPC patients

To study the significance of leptin in NPC, we first assessed its RNA level in NPC tissues from 20 patients diagnosed with NPC. Compared to benign nasopharyngeal inflammatory tissues, we observed that leptin mRNA level was dramatically upregulated in NPC tissues (Fig. 1a). Consistently, elevated leptin mRNA was also observed in Oncomine HNC database (Supplementary Figure S1a-d). Accumulating evidence demonstrated that leptin could be detected in the extracellular space, indicating that it may be a biomarker in patients with NPC. Therefore, we evaluated the serum level of leptin in our study cohort of 20 patients with NPC and 17 healthy controls. Results showed that the serum level of leptin in patients with NPC was significantly higher compared to that of healthy controls (Fig. 1b).

To explore the clinical significance of leptin in patients with NPC, IHC was performed in 50-paired paraffin-embedded NPC tumor tissues and adjacent non-tumor tissues. As shown in representative IHC images, leptin was primarily located in the cytoplasm in both adjacent non-tumor tissues and NPC tissues. In addition, it was highly expression in the NPC tissues than in the adjacent non-tumor tissues. Noticeably, strong leptin expression was positively correlated with advanced primary tumor (T) stage and neck metastatic lymph node (N) stage in NPC tumor tissues (Fig. 1c). Next, to explore the correlation between leptin expression and clinicopathological characteristics and the outcomes of patients with NPC, the intensities of IHC staining in NPC tumor tissues were divided into a high- or low- expression group. As shown in Table 1, statistical analysis revealed that high leptin levels were associated with T, N, and AJCC stage (*p* < 0.001) but not with pre-radiotherapy body mass index (pre-RTO BMI) (Table 1). In addition, survival analyses revealed that high leptin expression significantly correlated with a poor overall survival (OS) and disease-free survival (DFS) in patients with NPC (Fig. 1d and e). Collectively, these results demonstrated that leptin is a prognostic hallmark for NPC.

**Table 1:** Clinical characteristics of the NPC patients

Characteristics	N (%)/median (IQR)	Leptin		*p*-value
		Low (*n* = 28)	High (*n* = 22)	
Age	50(42–60)	54.5(45.3–61.0)	53.5(41.8–60.3)	0.615
Gender				
Female	13(26.0%)	9(32.1%)	4(18.2%)	0.264
Male	37(74.0%)	19(67.9%)	18(81.8%)	
AJCC stage				
I	8(16.0%)	8(28.6%)	0(0.0%)	< 0.001*
II	11(22.0%)	10(35.7%)	1(4.5%)	
III	7(14.0%)	6(21.4%)	1(4.5%)	
IV	24(48.0%)	4(14.3%)	20(90.9%)	
T stage				
1	14(28.0%)	13(46.4%)	1(4.5%)	< 0.001*
2	17(34.0%)	13(46.4%)	4(18.2%)	
3	1(2.0%)	0(0.0%)	1(4.5%)	
4	18(36.0%)	2(7.1%)	16(72.7%)	
N stage				
0	19(38.0%)	17(60.7%)	2(9.1%)	< 0.001*
1	12(24.0%)	3(10.7%)	9(40.9%)	
2	14(28.0%)	7(25.0%)	7(31.8%)	
3	5(10.0%)	1(3.6%)	4(18.2%)	
M stage				
0	25(78.1%)	13(81.3%)	12(75.0%)	1.000
1	7(21.9%)	3(18.8%)	4(25.0%)	
T stage				
1&2	31(62.0%)	26(92.9%)	5(22.7%)	< 0.001*
3&4	19(38.0%)	2(7.1%)	17(77.3%)	
N stage				
0	19(38.0%)	17(60.7%)	2(9.1%)	< 0.001*
1&2&3	31(62.0%)	11(39.3%)	20(90.9%)	
AJCC stage				
I & II	19(38.0%)	18(64.3%)	1(4.5%)	< 0.001*
III & IV	31(62.0%)	10(35.7%)	21(95.5%)	
Pre-RTO BMI	24.46(21.59–26.93)	24.17(19.97–27.06)	23.59(21.04–26.49)	0.749

*Abbreviations*: *NPC* nasopharyngeal carcinoma, *Pre-RTO BMI* pre-radiotherapy body mass index

*: *p*<0.05

### Leptin promotes NPC cell proliferation

**Fig. 2:**
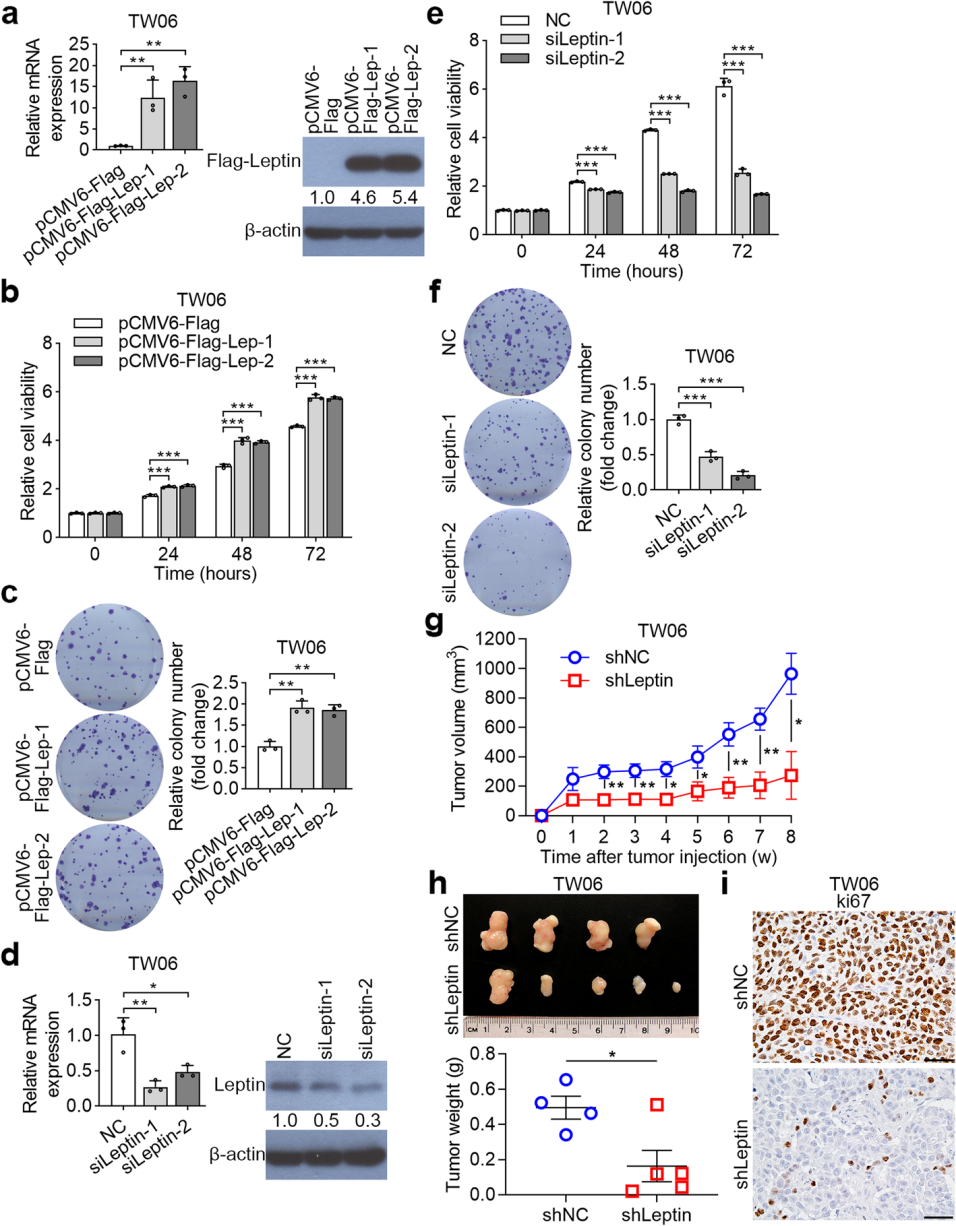
Leptin expression modulates the cell growth of NPC. **a** The mRNA and protein expression levels of leptin were assessed in TW06 cells by QPCR and Western blotting. **b** Cell growth ability was detected by MST assay over four consecutive days. Relative cell growth was normalized to day 0. **c** The colony formation assays evaluated the effect of leptin on cell proliferation. The representative images and fold change of foci formation were shown. **d** Leptin expression levels were detected by QPCR and Western blotting after transfection with either a negative control or leptin siRNA. **e** and **f** The effect of siLeptin-TW06 on cell proliferation was determined by MTS and colony formation assays. **g**-**h** shLeptin-TW06 or sh-NC-TW06 cells were injected into the right flank of null mice for 8 weeks. The tumor volumes were measured every week. The tumor weight in each group was calculated. **i** Representative images of Ki67 staining were conducted to demonstrate the proliferative cells. *P < 0.05, **P < 0.01, ***P < 0.001

To explore the role of leptin in NPC, we evaluated leptin expression levels in NPC cell lines. As shown in Supplementary Figure S1e, leptin mRNA and protein were extensively expressed in all NPC cell lines. Both TW06 and TW02 cell lines were chosen for analyzing the biological functions of leptin. We explored the effects of enhanced leptin expression by transfecting an ectopic-expressing vector into TW06 and TW02 cell lines. The mRNA and protein expressions of leptin in transfected cell lines were determined using QPCR and Western blotting (Fig. 2a and Supplementary Figure S2a). Thereafter, MTS and colony formation assays were performed, which showed that enforced leptin significantly increased cell proliferation capacity and colony formation ability compared to Flag-vector alone (Fig. 2b, c and Supplementary Figure S2b and S2c).

To further validate the results of gain-of-function of leptin, we transfected TW06 and TW02 cells with leptin siRNA or negative control. The transfection efficiencies of these two cell lines were determined using QPCR and Western blotting (Fig. 2d and Supplementary Figure S2d). Consistently, knockdown of leptin using shleptin decreased endogenous leptin mRNA and protein expression levels in TW06 cells (Supplementary Figure S2e). Furthermore, sileptin-mediated knockdown in TW06 and TW02 cells dramatically inhibited cell proliferation and foci formation (Fig. 2e, f and Supplementary Figure S2f and S2g). The effect of leptin on cell growth was further validated in a xenograft model. Tumors were found in 5/5 and 4/5 of mice injected with shleptin-TW06 and sh-NC-TW06 cells, respectively. Tumors derived from shLeptin-TW06 cells were much smaller and lighter than those in the sh-NC group. The proliferation index in tumors, determined by Ki67 staining, was attenuated with leptin knockdown (Fig. 2g-i). Taken together, leptin plays an important role in the growth of NPC cells.

### Leptin contributes to NPC cell migration and invasion by promoting epithelial-to-mesenchymal transition (EMT)

**Fig. 3:**
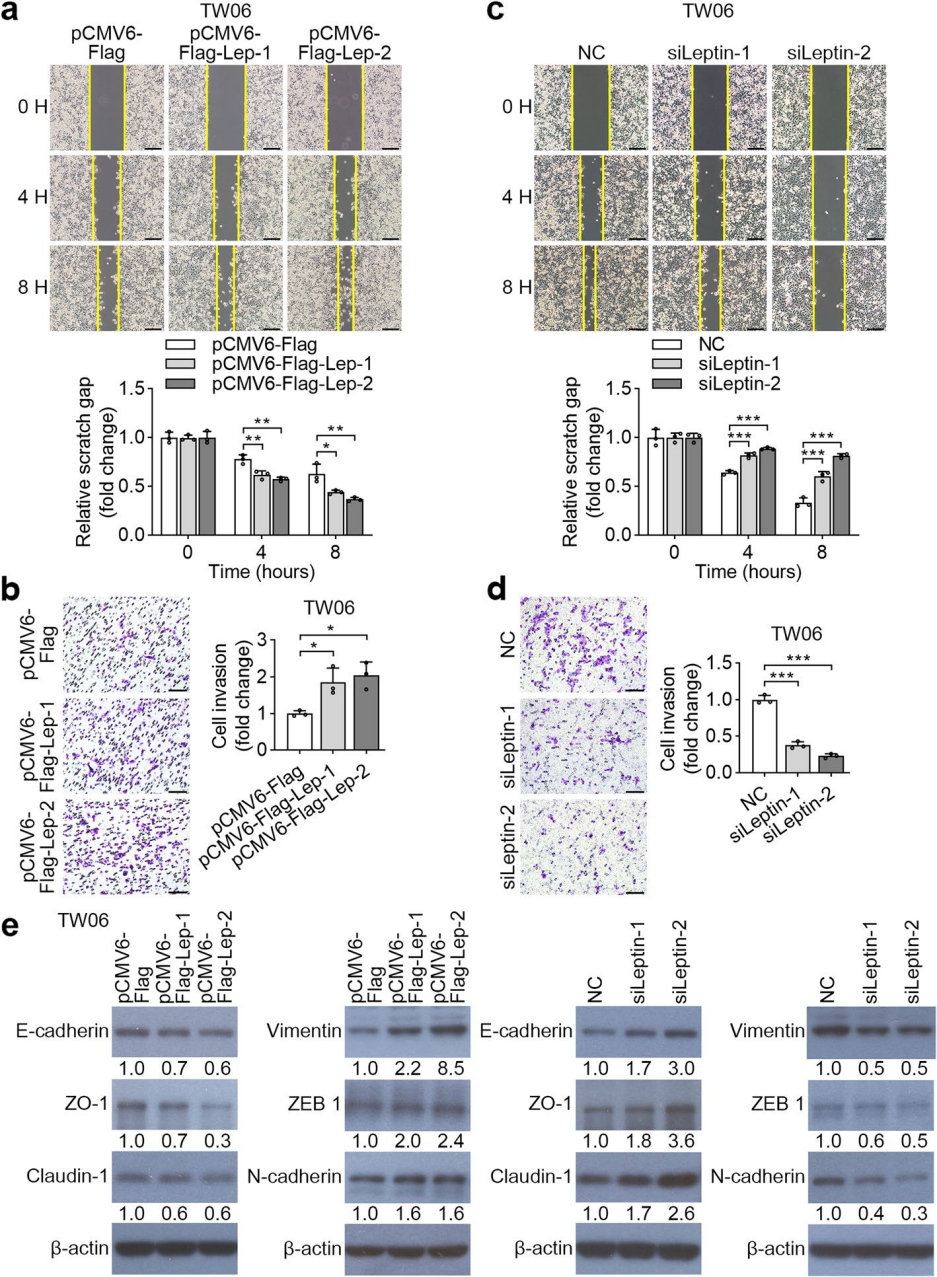
Leptin facilitates cell migration and invasion in NPC through EMT. **a** and **c** Wound healing assays were conducted to detect change in migratory ability in gain- or loss-of-function of leptin in TW06 cells. **b** and **d** TW06 cells with leptin overexpression or silence were subjected to Transwell assays. The representative images and the fold change of cell invasion were presented. **e** Expressions of EMT markers were detected by Western blotting in gain- or loss-of-function of leptin in TW06 cells. *P < 0.05, **P < 0.01, ***P < 0.001

Our clinical data showed that leptin expression was positively associated with metastatic features in patients with NPC, indicating that leptin may participate in cancer motility. To test this hypothesis, we first examined whether leptin expression had an impact on cell migration and invasion. Wound healing assays showed that enforced leptin expression significantly promoted cell migration in TW06 and TW02 cells as compared to vector control (Fig. 3a and Supplementary Figure S3a). In contrast, silencing leptin by two siRNAs in TW06 and TW02 dramatically attenuated cell migration (Fig. 3c and Supplementary Figure S3c). Furthermore, Transwell assays revealed that leptin overexpression enhanced the invasiveness of TW06 and TW02 cells (Fig. 3b and Supplementary Figure S3b), whereas leptin depletion inhibited cell movement in NPC cells (Fig. 3d and Supplementary Figure S3d). These results indicated that leptin might be involved in the metastasis of NPC tumors. In cancer, EMT is associated with tumor invasion, and metastasis. Herein, we investigated the expression profiles of EMT-related markers in gain- and loss-of-function of leptin by Western blotting. As shown in Fig. 3e and Supplementary Figure S3e, compared with vector alone, vimentin, ZEB1, and N-cadherin were upregulated and E-cadherin, ZO-1 and Claudin-1 were downregulated by leptin-overexpression in both TW06 and TW02. Conversely, leptin depletion displayed a reversed trend in the expression of EMT markers. Taken together, these data demonstrated that leptin triggers the EMT process in NPC cells to promote cell motility.

### Metabolic profiling is altered with leptin expression in NPC

**Fig. 4:**
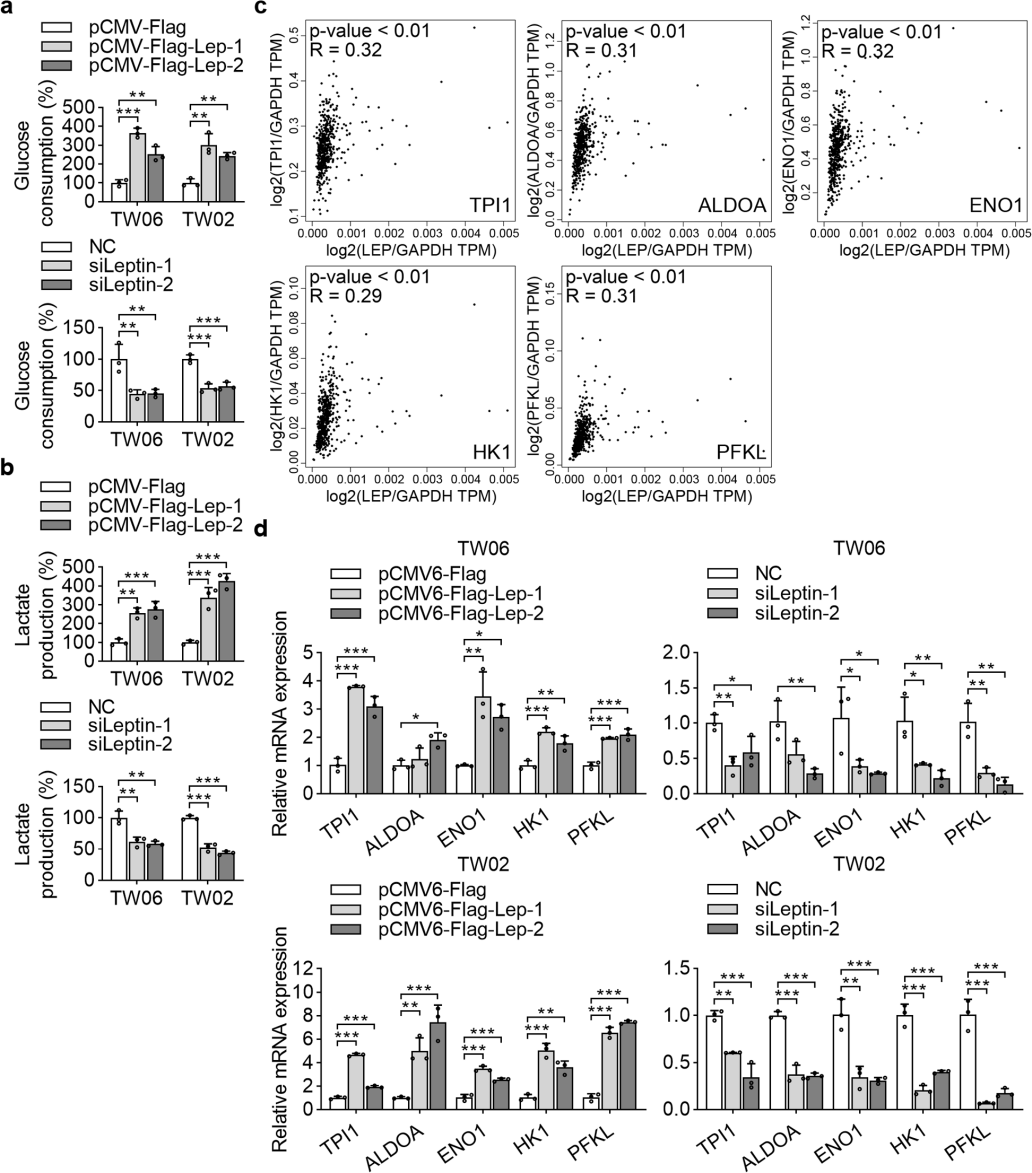
Leptin potentiates glycolysis in NPC cells. **a** and **b** The glucose consumption and lactate production were measured in NPC cells with gain- or loss-of-function of leptin. **c** The association of leptin and glycolytic molecules in GEPIA database was analyzed. **d** The transcriptional profiles of glycolytic molecules were examined in leptin-overexpressed and leptin-depleted NPC cell lines by QPCR. *P < 0.05, **P < 0.01, ***P < 0.001

Metabolic reprogramming is considered a hallmark of cancer and contributes to cancer progression [32]. Cancer cells prefer aerobic glycolysis to provide sufficient energy for cell growth. In this regard, we evaluated whether the observed proliferative effect of leptin occurred in association with glycolytic activity. The data showed that overexpression of leptin led to increased glucose consumption and lactate production in the culture medium of NPC cells (Fig. 4a and b). Conversely, leptin knockdown decreased glucose consumption and lactate production (Fig. 4a and b).

We next investigated if glycolysis-related genes were regulated by leptin. We systematically analyzed the correlation between leptin and multiple glycolytic genes via the GEPIA database, and the results indicated that the mRNA level of leptin was positively correlated with mRNA expressions of TPI1, ALDOA, ENO1, HK1 and PFKL (Fig. 4c). Ectopic overexpression of leptin in TW06 and TW02 cells induced the transcript levels of TPI1, ALDOA, ENO1, HK1 and PFKL. However, inhibition of endogenous leptin in NPC cells had the opposite effects (Fig. 4d). Altogether, leptin regulates multiple transcripts to directly promote glycolysis in NPC cells.

### Cell cycle-related proteins and EGFR/MAPK/c-Myc pathways are regulated by leptin in NPC

**Fig. 5:**
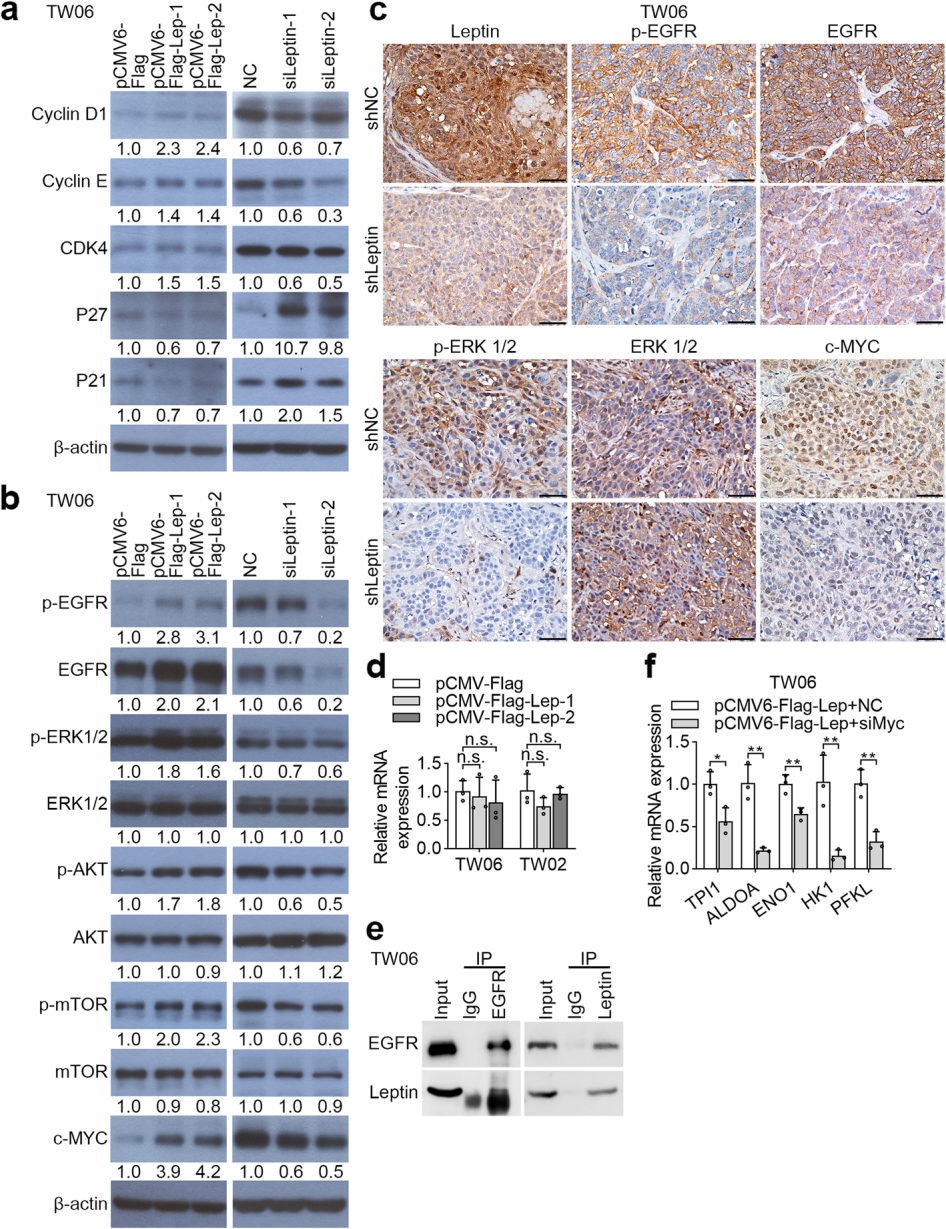
Leptin triggers cell cycle-related protein expression and EGFR/MAPK/c-Myc pathway in NPC. **a** Western blot indicated the expressions of cyclin D1, cyclin E, CDK4, p21 and p27 in leptin-overexpression and leptin-depleted TW06 cells. **b** Western blot analysis was performed to detect the protein levels of p-EGFR, EGFR, p-ERK1/2, ERK1/2, p-AKT, AKT, p-mTOR, mTOR and c-Myc in leptin-overexpression and leptin-depleted TW06 cells. **c** The IHC was performed to determine the protein expressions of leptin, p-EGFR, EGFR, p-ERK, ERK and c-Myc in the tumor tissues from shleptin-bearing xenograft model. **d** The EGFR mRNA levels were determined in gain-of-function of leptin in NPC cell lines. **e** TW06 cell lysates were subjected to EGFR immunoprecipitation with an anti-EGFR antibody or a control antibody, or subjected to leptin immunoprecipitation with an anti-leptin antibody, followed by Western blot of immunoprecipitation with an anti-leptin antibody or with an anti-EGFR antibody, respectively. **f** The transcriptional profiles of glycolytic molecules were examined by QPCR in leptin-overexpressed TW06 cell lines with c-Myc knockdown. n.s. no significant; *P < 0.05, **P < 0.01

Next, we considered the mechanism underlying the oncogenic role of leptin. The abnormality of cell cycle is a key mechanism for tumor development. Both cyclin D1 and cyclin E (form G1 to S phase) play key roles in the regulation of cell cycle and are important targets for cancer cell proliferation [33]. Hence, we determined whether leptin regulated both cyclin protein expressions in NPC cell lines. We observed prominent upregulation of cyclin D1, cyclin E and cyclin-associated protein CDK4 in TW06 and TW02 with leptin overexpression. However, two main members of negative regulators of cell cycle, p21 and p27 were down regulated with leptin overexpression (Fig. 5a and Supplementary Figure S4a). In contrast, leptin silencing caused down-regulation of cyclin D1, cyclin E, and CDK4, but up-regulation of p21 and p27 in both TW06 and TW02 cells (Fig. 5a and Supplementary Figure S4a). Collectively, leptin altered cell-cycle progression to influence cell proliferation in NPC cells.

A recent study reported that EGFR signaling is activated in human NPC and plays a crucial role in NPC pathogenesis. We next assessed if EGFR signaling was regulated by leptin in NPC. Western blotting showed that phosphorylated EGFR, phosphorylated ERK1/2, phosphorylated AKT, and phosphorylated mTOR, and c-Myc, an EGFR downstream target, were increased in leptin-overexpressed cells, and reduced in leptin-knockdown cells (Fig. 5b and Supplementary Figure S4b). These results were also confirmed in xenograft tumor samples by IHC staining (Fig. 5c). However, the QPCR analysis revealed that leptin overexpression in NPC cell lines did not influence EGFR mRNA level (Fig. 5d), indicating that leptin may regulate EGFR level post-transcriptionally. We then investigated if leptin and EGFR could physically interact by performing co-immunoprecipitation experiments in TW06 cells. Interestingly, an interaction between endogenous leptin and endogenous EGFR was observed in reciprocal co-immunoprecipitation assays (Fig. 5e). In addition, we also found that the transcript levels of glycolysis-related genes in leptin-overexpressing TW06 cells with c-Myc downregulation were decreased, compared to leptin-overexpression cells transfected with negative control (Fig. 5f). Collectively, leptin promotes NPC progression through binding with EGFR, thereby enhancing EGFR/MAPK/c-Myc activation.

### Leptin is a direct target of miR-874-3p

**Fig. 6:**
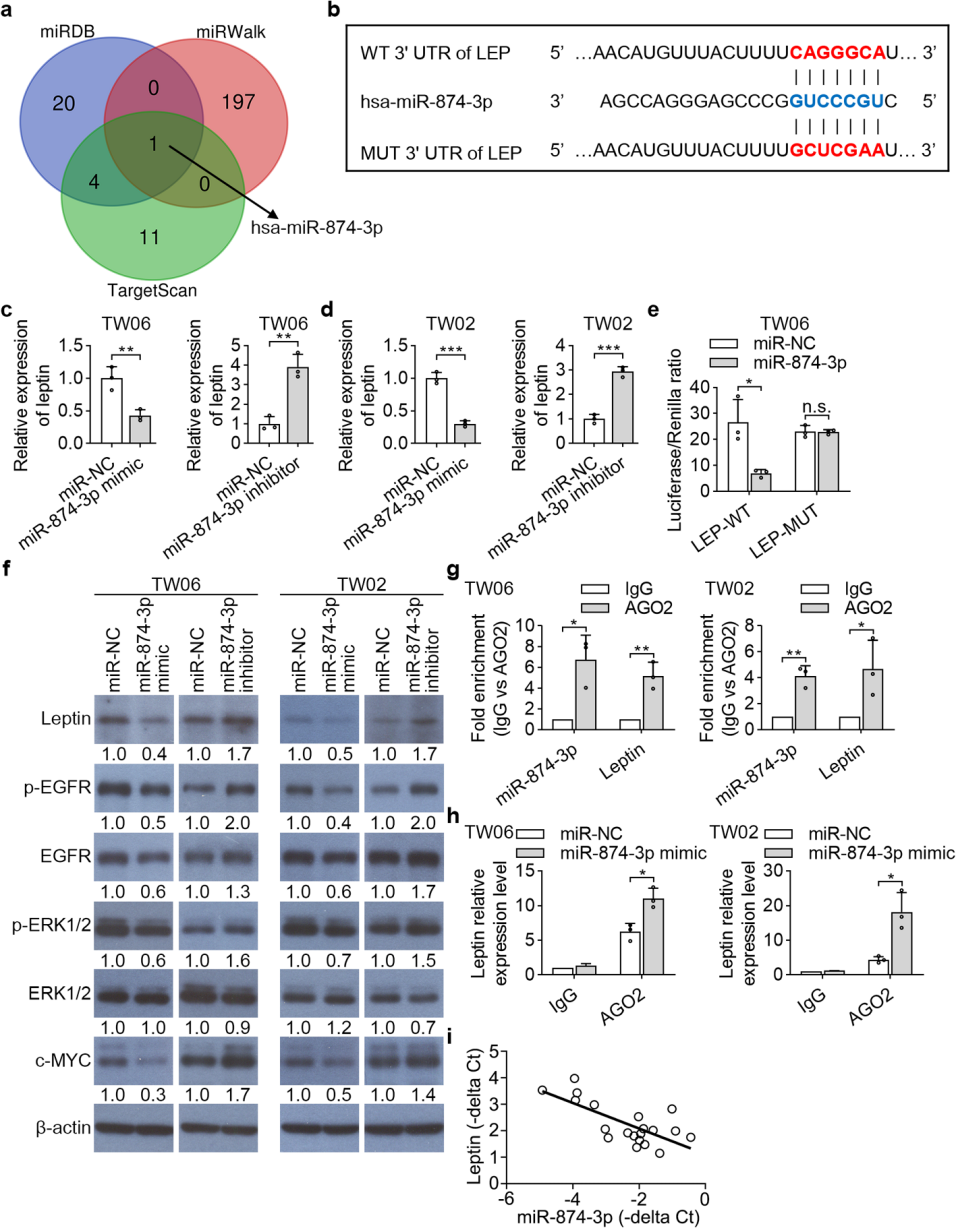
Leptin is targeted by miR-874-3p. **a** Venn diagram of putative miRNA targeting to leptin, and target prediction software miRDB, miRWalk and TargetScan were used for this study. **b** Predicted binding sites of miR-874-3p within the 3’UTR of leptin mRNA. **c** and **d** TW06 and TW02 cells were transfected with miR-874-3p mimics or miR-874-3p inhibitor for 48 h. The leptin mRNA expression levels were examined by QPCR. **e** Luciferase reporter assays were performed to determine the effect of miR-874-3p on the activity of leptin 3’UTR. **f** Western blot analysis of leptin and leptin downstream molecules in NPC cells transfected with miR-874-3p mimic, or miR-874-3p inhibitor, and corresponding negative control (NC). **g** The RIP assay was performed using AGO2 or IgG antibodies in NPC cells and the enrichment of miR-874-3p and leptin were detected by QPCR. **h** The RIP assays were also performed using AGO2 or IgG to estimate the enrichment of leptin in NPC cells transfected with miR-874-3p mimics and negative control. **i** The mRNA level of leptin was negatively correlated with miR-874-3p in NPC samples. *P < 0.05, **P < 0.01, ***P < 0.001

miRNA expression profiling affects the function of genes involved in tumorigenesis, proliferation, migration, and invasion in human cancers [34]. To identify possible miRNAs that directly regulate leptin expression, we searched miRNA databases that have putative binding sites in the 3'UTR of human leptin mRNA. One miRNA, has-miR874-3p, was predicted by three bioinformatic algorithms (miRDB, miRWalk and TargetScan) to be a potential upstream regulator of leptin (Fig. 6a). A putative binding site between miR-874-3p and leptin mRNA was shown in Fig. 6b. As expected, the mRNA and protein levels of leptin were decreased in TW06 and TW02 cells following the ectopic expression of miR-874-3p and increased by the miR-874-3p inhibitor (Fig. 6c, d and f). To determine whether miR-874-3p directly suppresses leptin expression by targeting its 3'UTR, a luciferase reporter plasmid containing 3'UTR of leptin was constructed and the plasmids containing the mutation sequences of 3'UTR of leptin (mut) were used as negative control. The results showed that NPC cells transfected with miR-874-3p markedly inhibited the activity of wild type leptin 3’UTR but not mutant 3’UTR (Fig. 6e). RNA immunoprecipitation (RIP) experiments were performed based on AGO2 enrich in targets bound to miRNAs on the lysates of TW06 and TW02 cells using an antibody against AGO2. The results showed that the expression levels of miR-874-3p and leptin were enriched in AGO2-conjugated beads relative to IgG control group (Fig. 6g). Furthermore, in TW06 and TW02 cells transfected with miR-874-3p mimic increased in the enrichment of leptin transcript pulled down by AGO2, compared to negative control mimics through RIP assay (Fig. 6h). These findings provide evidence that miR-874-3p associates with AGO2 protein to form an RNA-induced silencing complex (RISC) in NPC cells. Importantly, the results of Western blotting confirmed that EGFR signaling molecules, regulated by leptin as shown in Fig. 5, were decreased in the NPC cells transfected with miR-874-3p mimic, but increased in those transfected with miR-874-3p inhibitor (Fig. 6f). In NPC samples, the leptin mRNA was reversely associated with miR-874-3p expression (Fig. 6i). Altogether, these results demonstrated that leptin is a downstream target of miR-874-3p, which modulates leptin expression in NPC.

### miR-874-3p inhibits the malignant properties of NPC cells in vitro

**Fig. 7:**
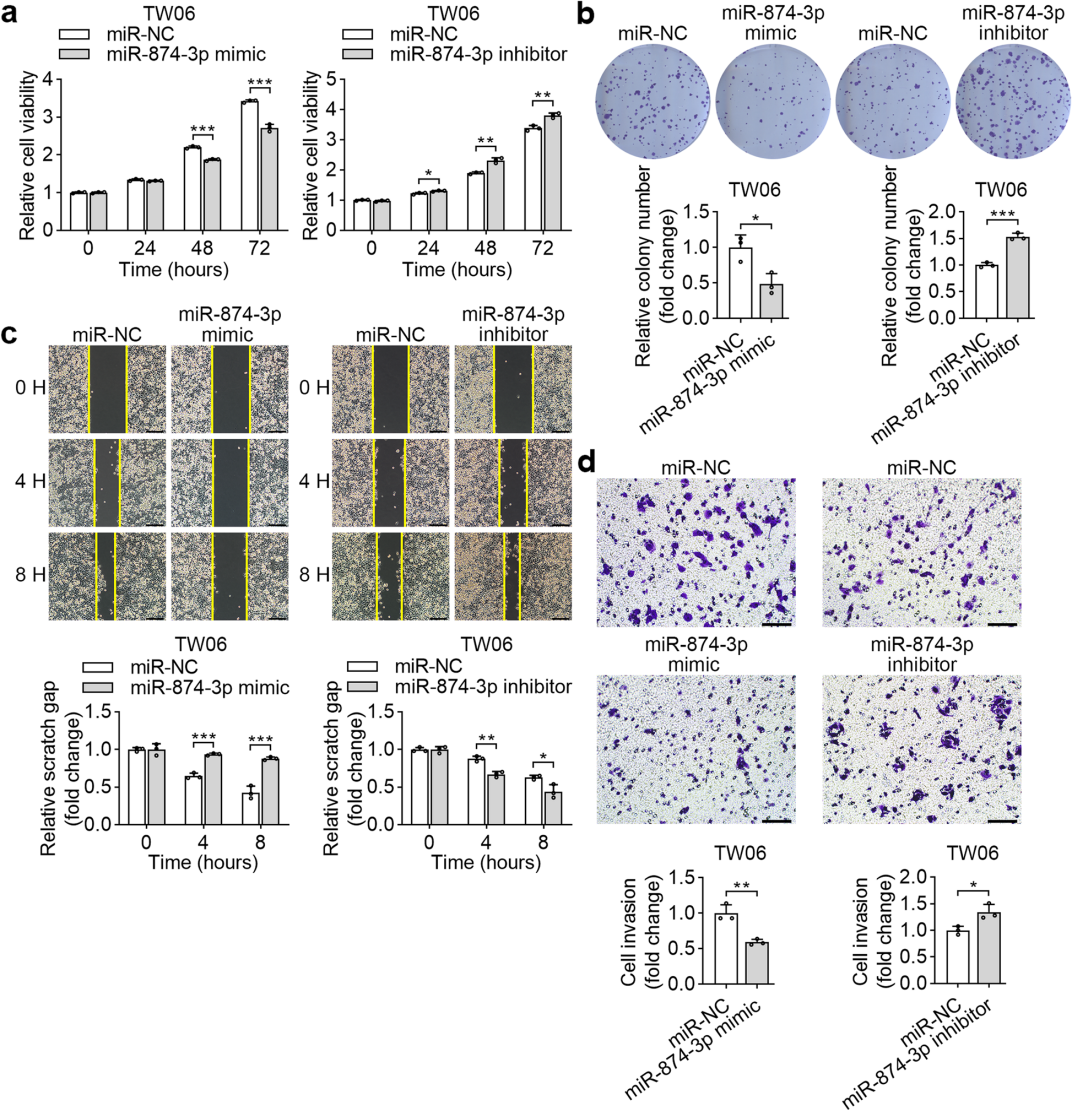
miR-874-3p suppresses the malignant properties of TW06 cells. **a** and **b** MTS assays and colony formation assays were performed to assess the cell proliferation of TW06 cells after transfecting miR-874-3p mimics or miR-874-3p inhibitor and their corresponding negative control. The representative images and fold change of foci formation were shown. **c** and **d** The migratory and invasive abilities of TW06 cells transfected with miR-874-3p mimics or miR-874-3p inhibitor and their corresponding negative control were assessed by wound healing and Transwell assays. *P < 0.05, **P < 0.01, ***P < 0.001

We further estimated the effects of miR-874-3p in various malignant properties of human NPC cell lines by transfecting with miR-874-3p mimic or miR-874-3p inhibitor. Introduction of miR-874-3p mimic in TW06 and TW02 cells significantly inhibited the proliferation and colony formation of cells compared to negative control mimic (Fig. 7a, b and Supplementary Figure S5a and b). Wound healing assay revealed that miR-874-3p mimic remarkably attenuated NPC cell migration as compared to control. However, overexpression of miR-874-3p inhibitor in NPC cells had the opposite effects (Fig. 7c and Supplementary Figure S5c). Transwell assays further revealed that the enforced miR-874-3p mimic significantly decreased the invasion capacity of NPC cells but increased it in NPC cells transfected with miR-874-3p inhibitor (Fig. 7d and Supplementary Figure S5d). These results suggest that miR-874-3p functions as a tumor suppressor in NPC.

### miR-874-3p restrains NPC cell growth, motility, and glycolytic activity by targeting leptin

**Fig. 8:**
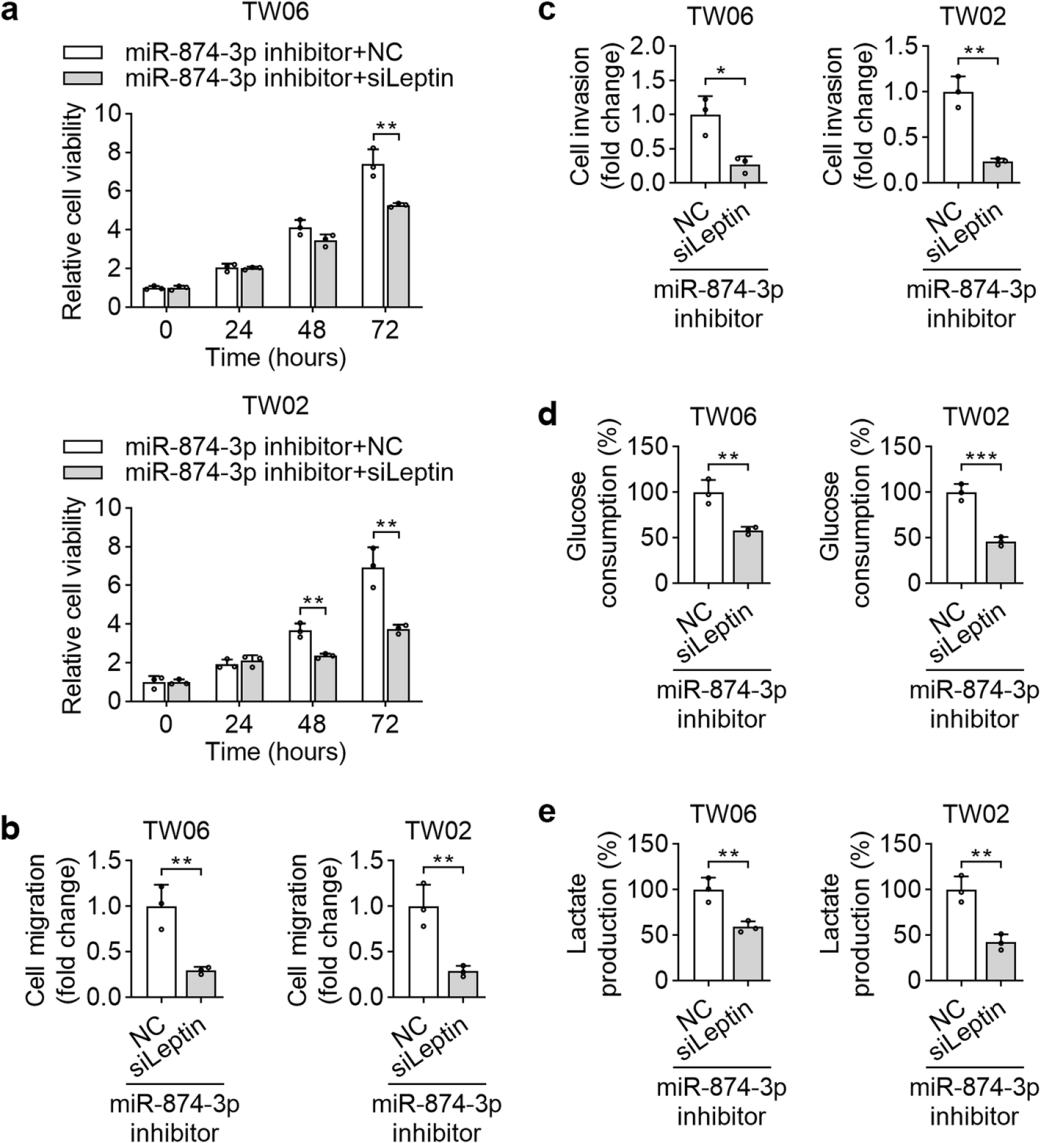
Leptin mediates the effects of miR-874-3p on NPC. NPC cells were cotransfected with sileptin or the siNC along with the miR-874-3p inhibitor to determine the abilities of cell growth (**a**), migration (**b**), invasion (**c**). The effects of miR-874-3p inhibitor on modulating glucose consumption (**d**) and lactate production (**e**) were also assessed by assay kits. *P < 0.05, **P < 0.01, ***P < 0.001

We performed a series of rescue experiments by cotransfecting the miR-874-3p inhibitor and sileptin to further explore the molecular mechanisms responsible for miR-874-3p-mediated NPC suppression. The impacts of miR-874-3p inhibitor on promoting the proliferation, migration, invasion, glucose consumption, and lactate production of NPC cells were substantially inhibited in cells transfected with sileptin (Fig. 8a to e). Overall, the downregulation of leptin at least partially contributes to the functional effects of miR-874-3p on NPC cells.

## Discussion

**Fig. 9:**
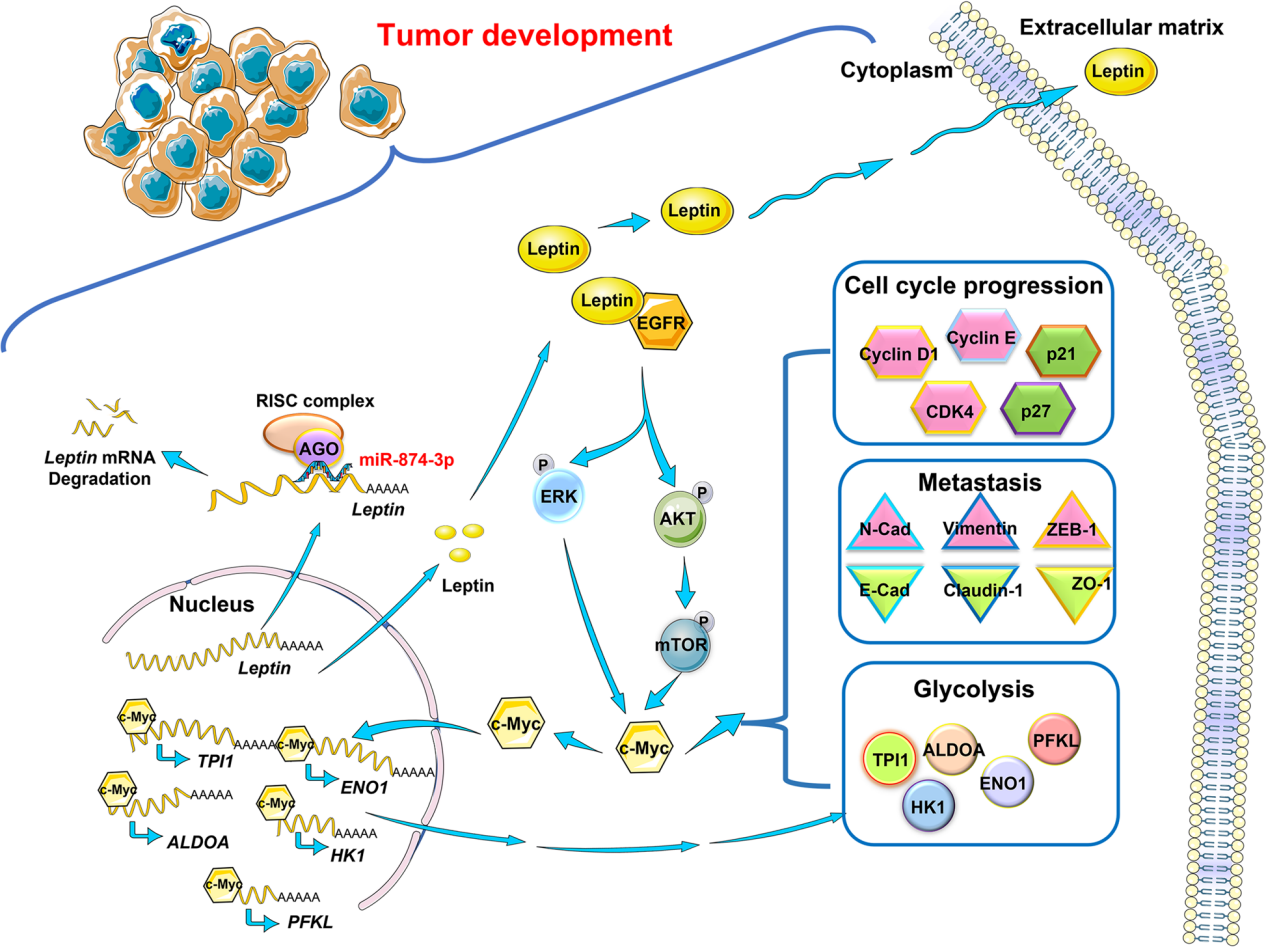
A proposed model for an unbalanced miR-874-3p/leptin/EGFR/c-Myc axis regulatory circuit in promoting NPC progression

In this study, we found that leptin was frequently overexpressed in NPC and associated with poor outcomes in patients with NPC. Functionally, leptin was expressed in all NPC cell lines and dramatically induced the growth, migration, invasion, and glycolysis of NPC cells. Conversely, silencing leptin exhibited the opposite effects. Moreover, leptin inhibition profoundly diminished the growth of xenografted NPC cells in vivo. The role of leptin in NPC was characterized as that of an oncogene. Interestingly, we also found that miR-874-3p is involved in leptin induced tumor progression in NPC. miR-874-3p expression suppressed proliferation, migration, invasion, glucose consumption and lactate production of NPC cells. Mechanistically, we demonstrated that leptin drove cancer development through EGFR signaling in NPC (Fig. 9). These finding reveal an important role of leptin in NPC progression and that it could be a potential therapeutic target for NPC.

Unlike cancers such as breast and colon cancers that have significant correlations between obesity and cancer risk [10, 13], studies on the correlations between obesity and NPC risk produced contradictory results [15, 16, 35–37]. In our study, although we did not observe a trend toward a higher BMI in patients diagnosed with NPC (data not shown), we found relatively high levels of circulating serum leptin and significant elevated mRNA and protein expression of leptin in nasopharyngeal tumor biopsies. Thus, leptin may play a critical role in NPC progression.

The elevated expression of leptin found within nasopharyngeal tumors (Fig. 1), raises the intriguing question of the potential causes of elevated intratumoral leptin expression. Although leptin is mainly expressed in adipose tissues, it has been detected in many other tissues/cells such as the brain, muscle, and stomach, as well as different types of cancer [38–42]. Despite the fact that there have been multiple studies revealing higher levels of leptin expression across many cancer tissues/cells [43, 44], the mechanistic details for the causes of these higher expression levels of leptin in cancer tissues/cells have only been addressed by a few studies [45]. By far, some of the mechanisms underlying the phenomenon of elevated intratumoral leptin expression have been investigated in breast and colorectal cancers [46]. These studies suggest that intratumoral leptin can be induced by hypoxic stress [46]. Thereby, it is possible that factors related to hypoxic stress may also contribute to the elevation of leptin expression in nasoharyngeal tumors. In fact, increased levels of factors related to hypoxic stress such as HIF-1α are often found in patients with NPC [47].

As mentioned previously*,* abundant expression of leptin, including its mRNA expression levels, have been found in several types of human cancer cells, as well as tumor biopsies [44]. Such expression is associated with poor prognosis and/or a higher risk of metastasis in patients with different cancers. For example, high leptin levels in tumor samples were related to recurrence of malignancy in laryngeal squamous cell carcinoma [44]. Elevated leptin mRNA levels were detected in tissue samples of patients with breast cancer [48]. Similarly, a study of non-small cell lung cancer (NSCLC) showed that higher leptin levels were detected in NSCLC tissues when compared to normal lung tissues [49]. This NSCLC study also further suggested that higher leptin levels in NSCLC samples were a predictor of poor prognosis [49]. In contrast to the aforementioned studies, negative leptin staining in breast cancer tumor samples is reportedly related to poor cancer survival [50]. Leptin expression levels also did not show any significant difference between the breast cancer tumor samples and control samples [50]. In gastro-oesophageal adenocarcinoma, tumors that had higher leptin expression were more chemoresistant compared to tumors with a lower leptin staining [40]. In addition, such high tumor leptin expression in gastro-oesophageal adenocarcinoma is associated with better survival [40]. In our study, we show that nasopharyngeal tumors are stained positively for leptin*.* Importantly, tumors that were from patients with more advanced stages of NPC (AJCC stages III /IV, T 3/4, N 1/2/3) tended to exhibit stronger staining of leptin. We also demonstrated that elevated intratumoral leptin is associated with a decreased DFS rate in patients with NPC with an estimated five-year DFS rate of only 27.3% (Fig. 2). To the best of our knowledge*,* our data provides the first evidence of correlations between elevated of intratumoral leptin and stages of cancer, as well as DSS rate in patients with NPC.

Distal-organ metastasis is commonly found in patients diagnosed with stage IV NPC and is largely responsible for the vast majority of deaths in patients with NPC. Therefore, it is important to understand the mechanisms of the spread of cancers cells from their primary tumors to distant parts of the body to more efficiently combat metastatic NPC. We showed that tumors with stronger leptin staining were collected from patients who were diagnosed with advanced stages III/IV of NPC. Furthermore, we also showed that leptin promotes NPC cell invasiveness, which is considered the very first step of metastasis. These data suggested that leptin may have a role in distal-organ metastasis in NPC. There is strong evidence that EMT is linked to metastasis of NPC [51]. However, it is unclear if the change of NPC cell invasiveness and elevated leptin in more advanced nasopharyngeal tumors that we observed in our experiments have a connection with EMT. To better understand if leptin confers potential advantages on metastatic NPC via EMT, we assessed the expression of EMT-related proteins.

EMT is typically known for its role in embryonic development but is now gaining recognition for its involvement in metastasis of tumors such as osteosarcoma, breast cancer and lung cancers [52]. Given that in the process of EMT, epithelial cancer cells disseminate as they lose their adhesive properties and expression of epithelial markers such a*s* E-cadherin, ZO-1 and Claudin-1, while gaining mesenchymal features and expression of mesenchymal markers such as vimentin, Zeb-1, and N-cadherin [52]. As a consequence, these disseminated cells undergo a phenotypic shift from epithelial to mesenchymal, which facilitates their migration from the primary tumors to distal parts of the body [52]. According to our results, altered levels of leptin in NPC cells impact the expression of some proteins involved in the EMT process. Leptin induces EMT by modulating the expression of EMT-related markers and promotes tumor metastasis [53, 54]. We also examined the effects of leptin on pathways such as the ERK and EGFR signaling pathways. Our data suggested that leptin is involved in alteration of diverse molecules related to EMT, ERK, and EGFR signaling pathways.

The abnormal metabolic programming has been found in human cancer cells. Cancer cells have higher glucose consumption and lactate production than normal cells [55]. Our data suggested that leptin influences the gene expression levels of glycolytic enzymes and thus alters glucose consumption and lactate production in NPC cells. Many studies have showed that c-Myc activates the transcription of all glycolytic genes directly through binding the classical E-box motif [56, 57]. In the present study, we demonstrated that the expression patterns of glycolytic genes were reduced in leptin overexpressing NPC cells with c-Myc knockdown. Collectively, these finding revealed that c-Myc plays an important role in regulation of glucose metabolism in NPC cells that may require for leptin. In addition, the expression patterns of these markers in response to leptin expression could possibly explain our findings of the association between elevated intratumoral expression of leptin and more advanced nasopharyngeal tumors and poor prognosis of NPC.

A growing body of evidence has reported that EGFR is highly expressed in 85% NPC patients, and its expression is correlated with poor outcome [58]. In vitro and in vivo studies have confirmed that high EGFR expression is associated with proliferation, migration, invasion of NPC cells [59, 60]. The mechanisms of EGFR overactivation in tumor development is mainly associated with EGFR. For instance, EGFR-AS1, a lncRNA, interacts with EGFR to promote cell growth and metastasis via upregulating EGFR expression in renal carcinoma [61]. SPINK6, a secreted protein, promotes metastasis of NPC by binding to EGFR and activating EGFR signaling pathway [60]. Moreover, as shown recently, Choline kinase a (CHKA), a catalytic enzyme for de novo biosynthesis of phosphatidylcholine could plays an indispensable role in the progression and metastasis of HCC through interacting with EGFR and enhancing mTORC2-dependent AKT pathway [62]. In the current study, we demonstrated that leptin binds to EGFR and facilitate the activation of EGFR signaling in NPC. Our results explored a novel EGFR-activating mechanism in which leptin has a critical role in NPC development.

Small RNAs, for example, miRNAs or siRNAs have revealed pivotal regulators of signaling pathways in cells. They can control gene expressions by suppression of translation or degradation of mRNAs. To degrade the target mRNAs, miRNA or siRNAs need to be loaded into the RNA-induced silencing complex (RISC) [63]. Argonaute (AGO) proteins are core components of the RISC and, are essential for small RNA biogenesis. In addition, AGO proteins have been found to be associated with miRNA or siRNA and involved in RNA silencing [63–65]. Thus, AGO proteins play important roles in small RNA biogenesis in mammalian cells. AGO family proteins are defined by the existence of PAZ and PIWI domains and can be broadly divided into two subgroups: the ubiquitously expressed AGO clade and germline-specific PIWI clade that binds piRNAs [66, 67]. In human, four AGO subfamily proteins, (AGO1-AGO4) and four PIWI subfamily protein (PIWI1-PIWI4), have been identified. All four AGO proteins can suppress miRNA target genes; however, AGO2 is the most important protein which has an enzymatically competent RNaseH-like domain and serves as an engine of the RISC to guide gene silencing processes [68]. In the current study, we provided evidence that miR-874-3p is associated with AGO2 protein to form an RISC in NPC cells, indicating that the miRNA pathway, in particular miR-874-3p, contributes to the gene regulatory axis in NPC.

Both siRNAs and miRNAs are short RNA duplexes but with distinct gene regulatory mechanisms. SiRNAs are involved in sequence-specific post-transcriptional gene silencing by binding to their target mRNAs, whereas, miRNAs are involved in multiple modes of post-transcriptional gene regulation by binding to their target mRNAs mostly at the 3'UTR site [69]. Both siRNAs and miRNAs are nonconding RNAs with great potential for developing targeted cancer therapies. Thus**,** we designed experiments to test the feasibility of targeting leptin as a therapeutic treatment for NPC using RNA-based therapeutic strategies such as those including siRNA and miRNA, in this study. Our data suggested that silencing leptin by either siRNA or miRNA reduces the gene expression of *leptin* mRNA and leads to the reduction of NPC cell survival and proliferation. Taken together, our results suggested that leptin is a potential therapeutic target for NPC and RNA-based strategies to target leptin are sufficient to reduce the effects of leptin on NPC carcinogenesis.

## Conclusions

In summary, we conducted a study to understand the effects of leptin on NPC by combining a small-scale clinical analysis, in vitro, in vivo and in silico methods. Our small-scale clinical findings indicated that elevated intra-tumoral leptin was associated with more advanced tumors and poor prognosis of NPC. Our data also suggested that leptin was involved in alteration of diverse molecules related to pathways that regulate EMT and cell proliferation. These results supported the idea that leptin facilitates nasopharyngeal cell survival, proliferation and invasion by influencing the expression levels of EMT-, cell proliferation- and glycolysis-related proteins, which are key to tumor invasiveness and metastasis and the shortened survival of patients with cancer. Similarly, these results may also explain the findings of our clinical research, as well as our mouse model of tumor growth, considering the cancer-promoting effects of leptin in our assays. Furthermore, we utilized in silico miRNA-target prediction to identify miR-874-3p as a novel regulator of leptin and rigorously validated this prediction experimentally. Our data suggests that miR-874-3p targets leptin mRNA to reduce cell survival and proliferation in NPC cells. Overall, this study provides insights into how leptin is involved in the carcinogenesis of NPC with a focus on potential pathological effects of leptin and therapeutic targeting of leptin expression, which may potentially be applied in future clinical settings.

## Supplementary Information

**Additional file 1:** **Supplementary Figure S1.** Leptin expression profiles are investigated in publicly database and NPC cell lines. (a to d) The mRNA expression levels of leptin in Oncomine HNC databases were determined. (e) Analysis of leptin mRNA and protein levels in NPC cell lines.

**Additional file 2:** **Supplementary Figure S2.** Leptin promotes cell growth in NPC. (a) The mRNA and protein expression levels of leptin were investigated in gain-of-function of leptin in TW02 cells by QPCR and Western blotting. (b) MTS assays was performed to assess the cell proliferation of TW02 cells after transfection of overexpression plasmid. (c) The foci numbers of TW02-leptin transfectants were assessed. The representative images and fold change of foci formation were shown. (d and e) The mRNA and protein expression levels of leptin were investigated in loss-of-function of leptin in TW02 and TW06 cells by QPCR and Western blotting. (f and g). The effect of siLeptin-TW02 on cell proliferation was determined by MTS and colony formation assays. *P<0.05, **P<0.01, ***P<0.001.

**Additional file 3:** **Supplementary Figure S3.** Leptin promotes NPC cells motility by inducing EMT. (a and c) Wound healing assays demonstrated that the overexpression or knockdown of leptin modulated the migratory ability of TW02 cells. (b and d) Transwell assays were performed to examine the change of invasive ability of TW02 cells with leptin overexpression or knockdown. (e) Expressions of EMT markers were detected by Western blotting in TW02 cells with overexpression or knockdown of leptin. *P<0.05, **P<0.01, ***P<0.001.

**Additional file 4:** **Supplementary Figure S4.** Leptin regulates the expressions of cell cycle-related molecules and EGFR/MAPK/c-Myc pathway in TW02 cells. (a) Western blot indicated the expressions of cyclin D1, cyclin E, CDK4, p21 and p27 in leptin-overexpression and leptin-depleted TW02 cells. (b) Western blot analysis was performed to detect the protein levels of p-EGFR, EGFR, p-ERK1/2, ERK1/2, p-AKT, AKT, p-mTOR, mTOR and c-Myc in leptin-overexpression and leptin-depleted TW02 cells.

**Additional file 5:** **Supplementary Figure S5.** miR-874-3p suppresses the malignant properties of TW02 cells. (a and b) MTS assays and colony formation assays were performed to assess the cell proliferation of TW02 cells after transfecting miR-874-3p mimics or miR-874-3p inhibitor and their corresponding negative control. The representative images and fold change of foci formation were shown. (c and d) The migratory and invasive abilities of TW02 cells transfected with miR-874-3p mimics or miR-874-3p inhibitor and their corresponding negative control were assessed by wound healing and Transwell assays. *P<0.05, **P<0.01, ***P<0.001.

## Data Availability

All data generated or analyzed during this study are included in this published article.

## Abbreviations

NPC: Nasopharyngeal carcinoma
EBV: Epstein-Barr virus
AJCC: American Joint Committee on Cancer
EMT: Epithelial-mesenchymal transition
QPCR: Quantitative polymerase chain reaction
TCGA: The Cancer Genome Atlas
