# Supplementary figures and images for: Aberrant miR-874-3p/leptin/EGFR/c-Myc signaling contributes to nasopharyngeal carcinoma pathogenesis
Source: J Exp Clin Cancer Res. 2022 Jul 1;41:215. doi: 10.1186/s13046-022-02415-0 (PMC9248092; doi:10.1186/s13046-022-02415-0)

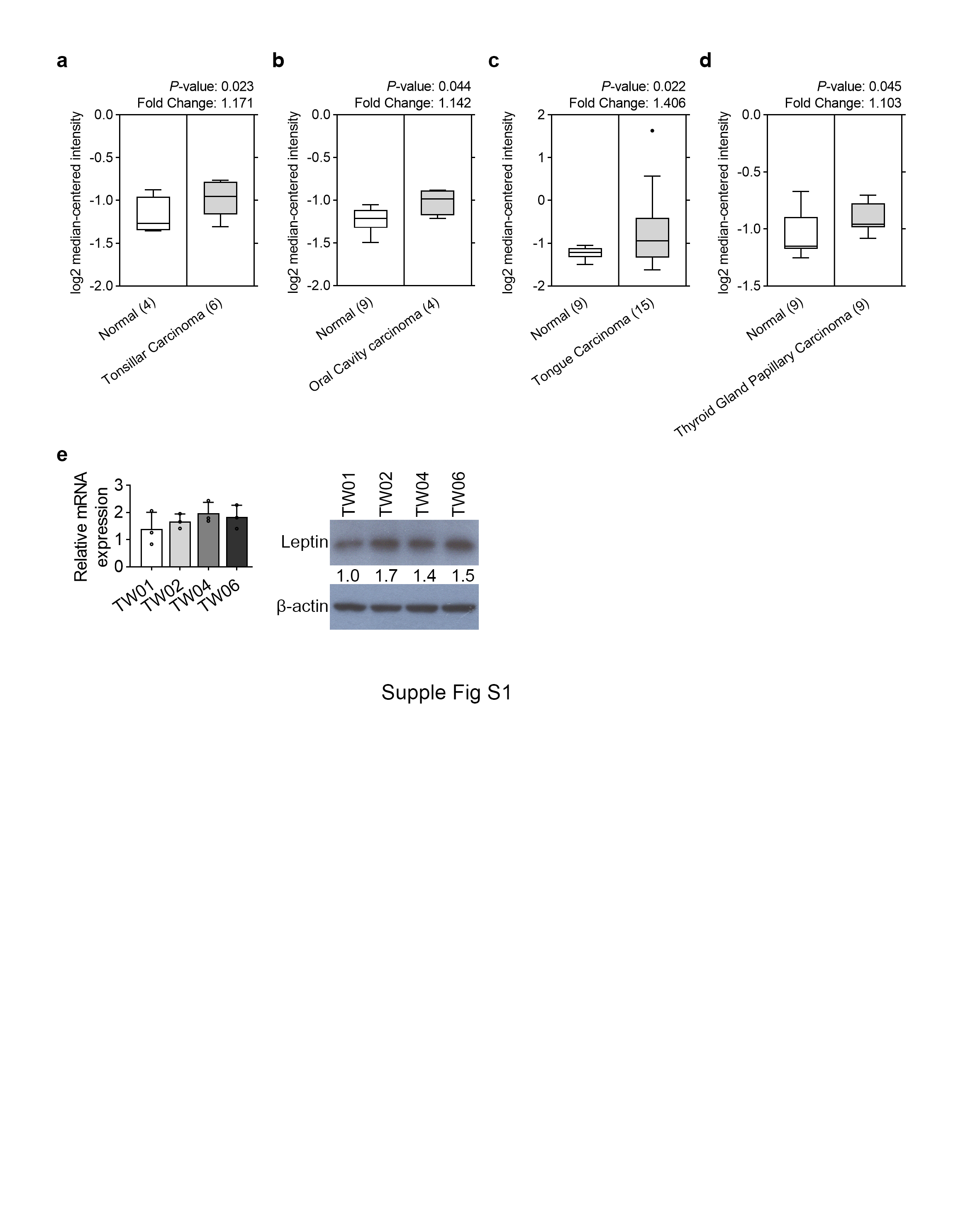

Supplement: Supplementary file 1 — Additional file 1: Supplementary Figure S1. Leptin expression profiles are investigated in publicly database and NPC cell lines. (a to d) The mRNA expression levels of leptin in Oncomine HNC databases were determined. (e) Analysis of leptin mRNA and protein levels in NPC cell lines. [file 13046_2022_2415_MOESM1_ESM.tif]

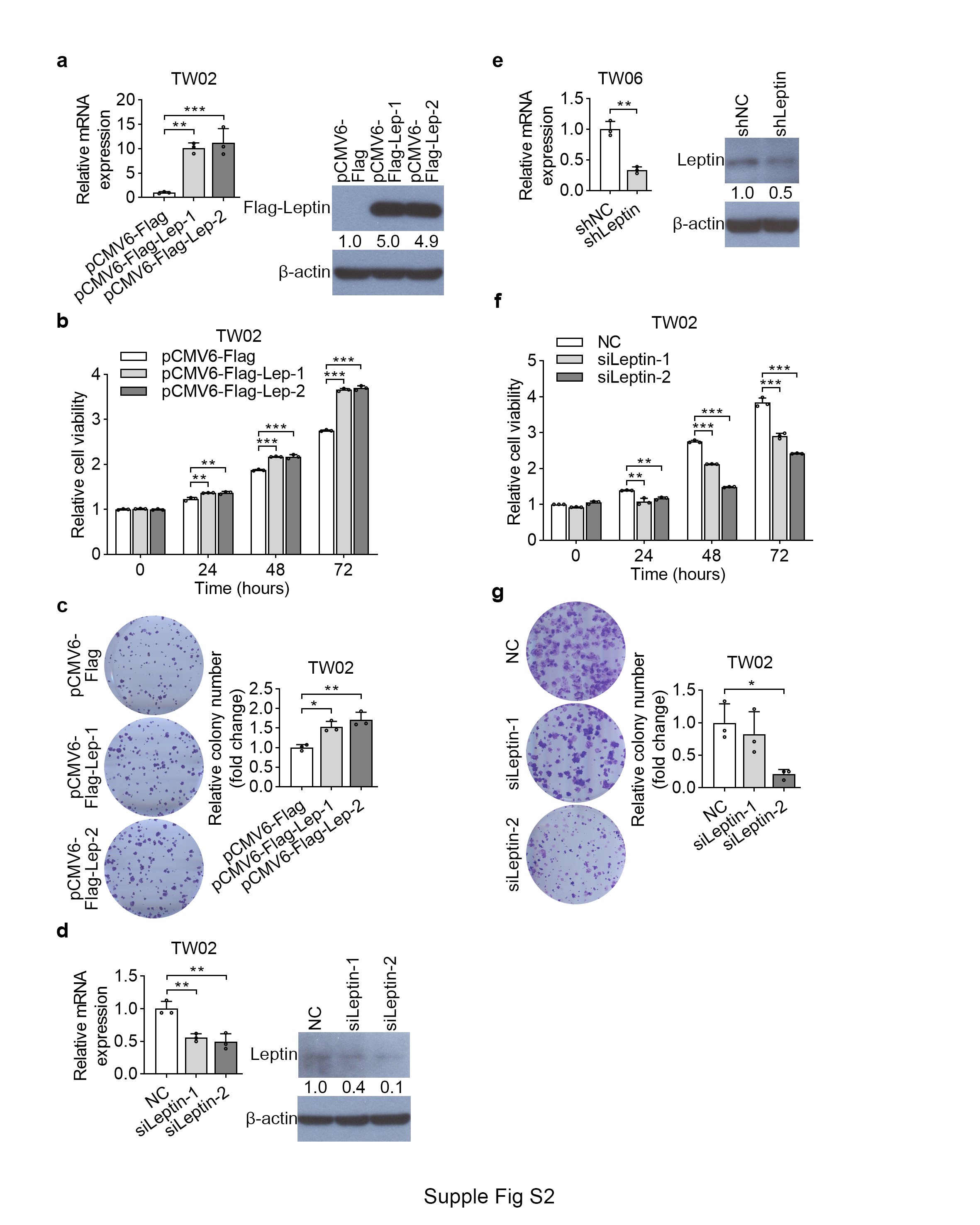

Supplement: Supplementary file 2 — Additional file 2: Supplementary Figure S2. Leptin promotes cell growth in NPC. (a) The mRNA and protein expression levels of leptin were investigated in gain-of-function of leptin in TW02 cells by QPCR and Western blotting. (b) MTS assays was performed to assess the cell proliferation of TW02 cells after transfection of overexpression plasmid. (c) The foci numbers of TW02-leptin transfectants were assessed. The representative images and fold change of foci formation were shown. (d and e) The mRNA and protein expression levels of leptin were investigated in loss-of-function of leptin in TW02 and TW06 cells by QPCR and Western blotting. (f and g). The effect of siLeptin-TW02 on cell proliferation was determined by MTS and colony formation assays. *P<0.05, **P<0.01, ***P<0.001. [file 13046_2022_2415_MOESM2_ESM.tif]

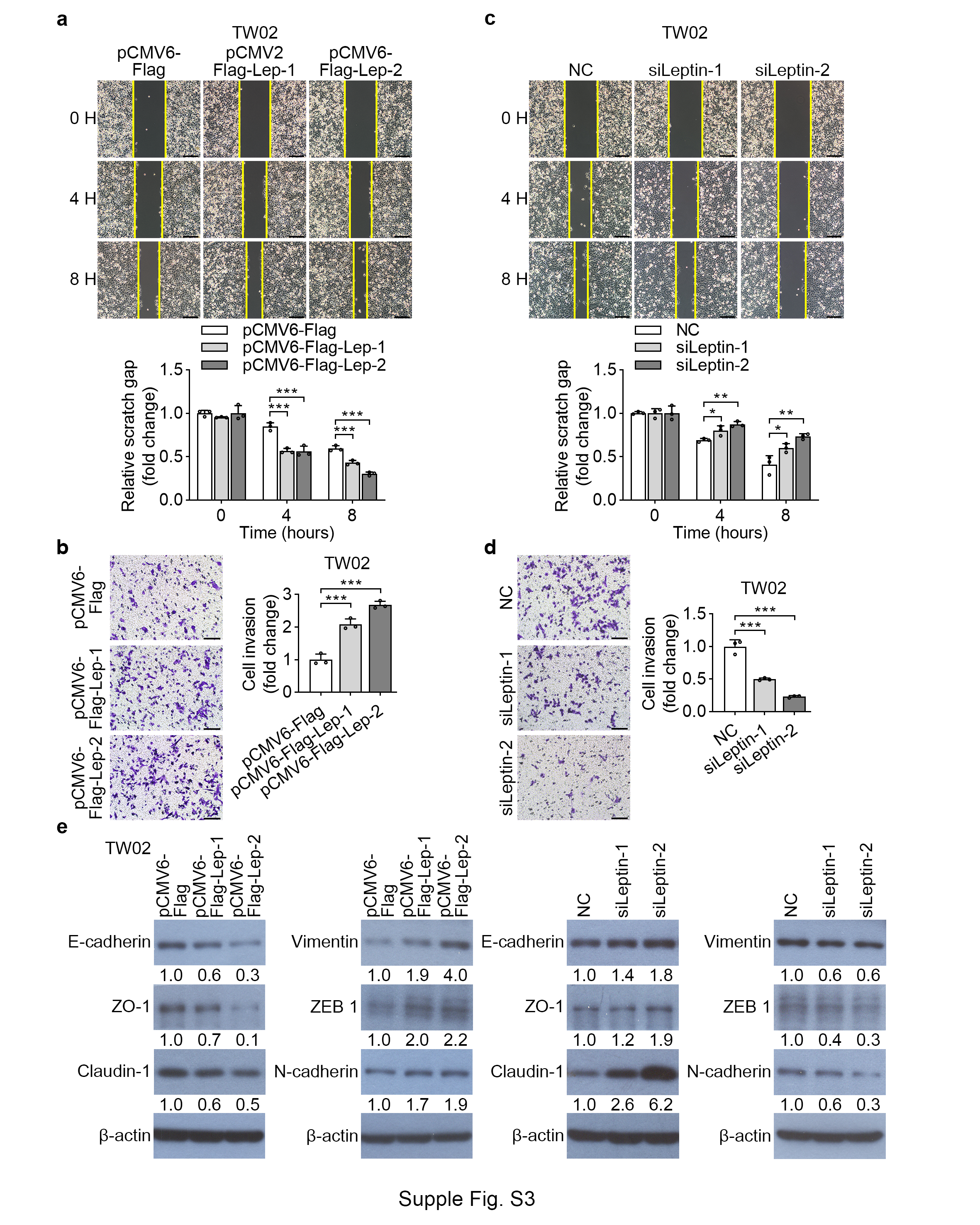

Supplement: Supplementary file 3 — Additional file 3: Supplementary Figure S3. Leptin promotes NPC cells motility by inducing EMT. (a and c) Wound healing assays demonstrated that the overexpression or knockdown of leptin modulated the migratory ability of TW02 cells. (b and d) Transwell assays were performed to examine the change of invasive ability of TW02 cells with leptin overexpression or knockdown. (e) Expressions of EMT markers were detected by Western blotting in TW02 cells with overexpression or knockdown of leptin. *P<0.05, **P<0.01, ***P<0.001. [file 13046_2022_2415_MOESM3_ESM.tif]

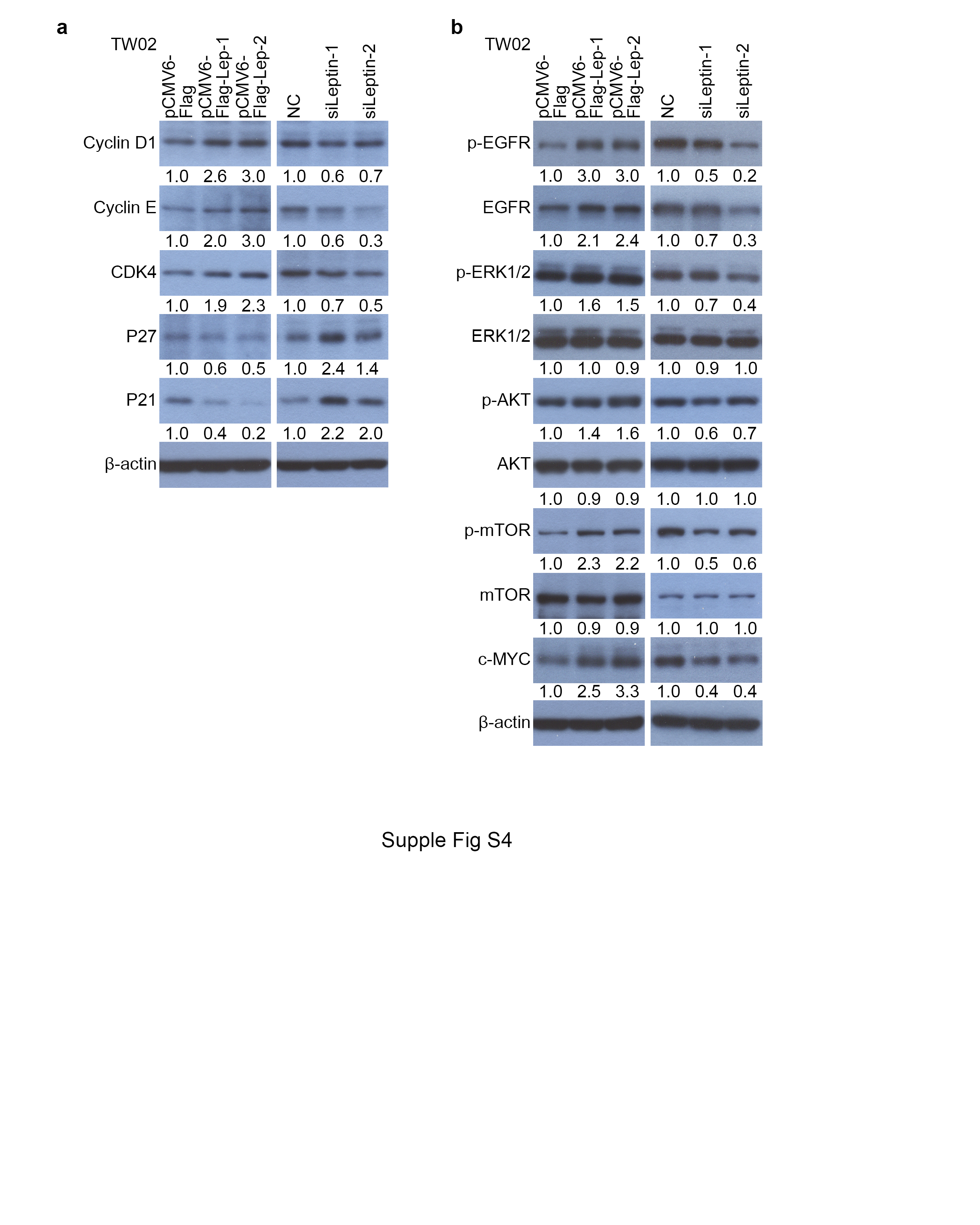

Supplement: Supplementary file 4 — Additional file 4: Supplementary Figure S4. Leptin regulates the expressions of cell cycle-related molecules and EGFR/MAPK/c-Myc pathway in TW02 cells. (a) Western blot indicated the expressions of cyclin D1, cyclin E, CDK4, p21 and p27 in leptin-overexpression and leptin-depleted TW02 cells. (b) Western blot analysis was performed to detect the protein levels of p-EGFR, EGFR, p-ERK1/2, ERK1/2, p-AKT, AKT, p-mTOR, mTOR and c-Myc in leptin-overexpression and leptin-depleted TW02 cells. [file 13046_2022_2415_MOESM4_ESM.tif]

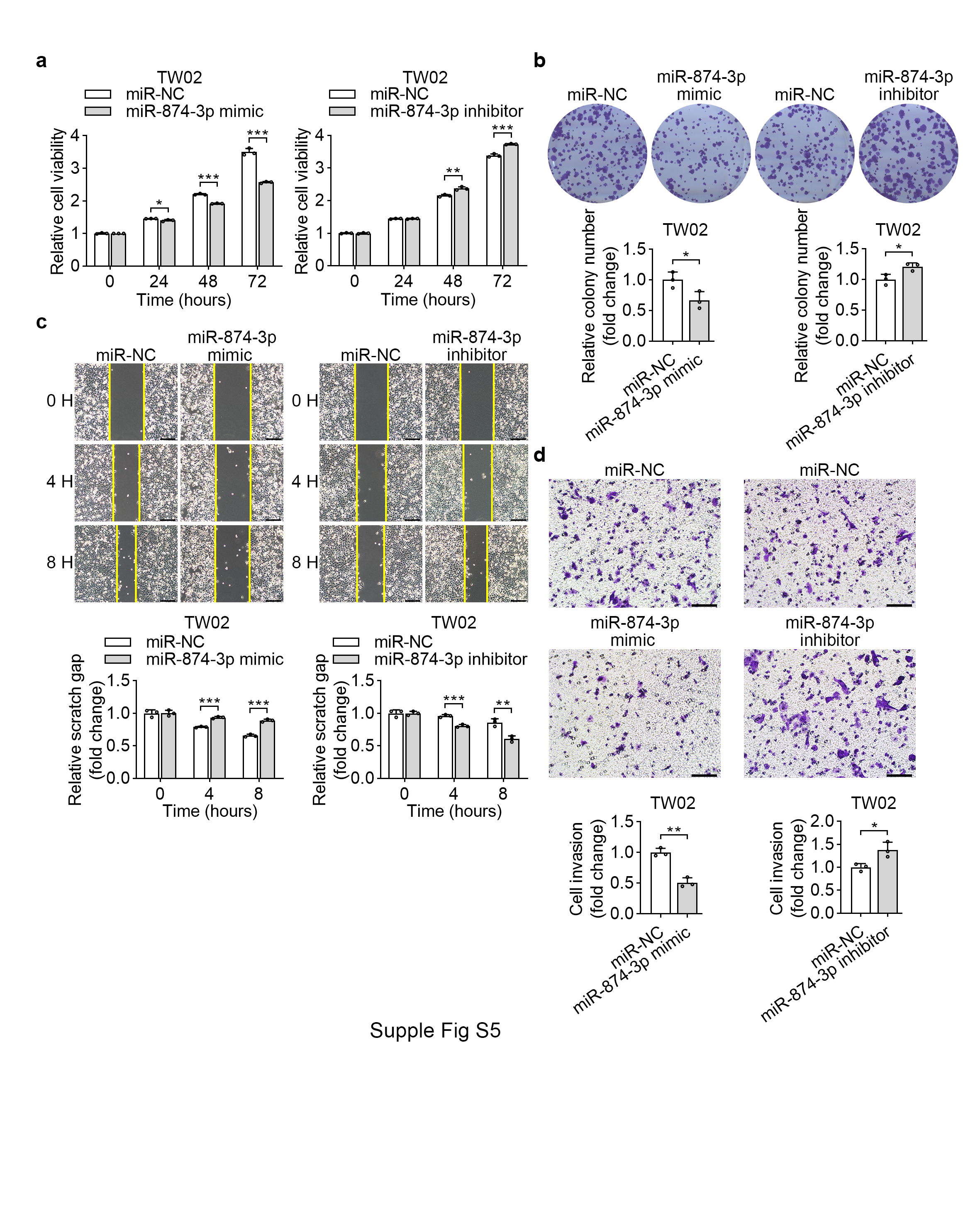

Supplement: Supplementary file 5 — Additional file 5: Supplementary Figure S5. miR-874-3p suppresses the malignant properties of TW02 cells. (a and b) MTS assays and colony formation assays were performed to assess the cell proliferation of TW02 cells after transfecting miR-874-3p mimics or miR-874-3p inhibitor and their corresponding negative control. The representative images and fold change of foci formation were shown. (c and d) The migratory and invasive abilities of TW02 cells transfected with miR-874-3p mimics or miR-874-3p inhibitor and their corresponding negative control were assessed by wound healing and Transwell assays. *P<0.05, **P<0.01, ***P<0.001. [file 13046_2022_2415_MOESM5_ESM.tif]
